# Pharmacist-Led Self-management Interventions to Improve Diabetes Outcomes. A Systematic Literature Review and Meta-Analysis

**DOI:** 10.3389/fphar.2017.00891

**Published:** 2017-12-14

**Authors:** Linda van Eikenhorst, Katja Taxis, Liset van Dijk, Han de Gier

**Affiliations:** ^1^Unit of PharmacoTherapy, -Epidemiology and -Economics, Groningen Research Institute of Pharmacy, University of Groningen, Groningen, Netherlands; ^2^Pharmaceutical Care, NIVEL, Netherlands Institute for Health Services Research, Utrecht, Netherlands

**Keywords:** diabetes, pharmacist, pharmacy practice, self-management, HbA1c, meta-analysis

## Abstract

**Background:** Treatment of diabetes requires a strict treatment scheme which demands patient self-management. Pharmacists are in a good position to provide self-management support. This review examines whether pharmacist-led interventions to support self-management in diabetes patients improve clinical and patient-reported outcomes.

**Methods:** This review was conducted according to the PRISMA guidelines. An extended literature search was conducted with the keywords “pharmacist,” “diabetes,” and “self-management” using the electronic databases Pubmed, Embase, CINAHL, PsycINFO, Web of Science, and the Cochrane Library from the beginning of the database through September 2017. In addition reference lists of systematic reviews and included studies were searched. Eligibility criteria included; self-management intervention tested with an RCT, performed in an ambulatory care setting, led by a pharmacist and reporting at least one clinical- or patient-reported outcome. Primary outcomes were HbA1c (—as this is a clinical parameter for long-term diabetes follow-up), self-management and components of intervention. Secondary outcomes were blood glucose, blood pressure, BMI, lipids, adherence to medication, quality of life, and diabetes knowledge. For the meta-analysis HbA1c values were pooled with a random-effects model in Revman 5.3. Risk of bias was assessed with the Cochrane Risk of Bias tool.

**Results:** Twenty-four studies representing 3,610 patients were included. Pharmacist-led self-management interventions included education on diabetes complications, medication, lifestyle, and teaching of self-management skills. Some studies focused on patient needs through a tailored intervention. No key components for a successful self-management intervention could be identified. Pharmacist-led self-management interventions improve HbA1c levels with a mean of 0.71% (CI −0.91, −0.51; overall effect *P* < 0.0001) and had a positive effect on blood pressure (SBP −5.20 mm Hg [−7.58; −2.92], DBP −3.51 mmHg [−6.00; −1.01]), BMI (−0.49 kg/m2 [−0.79; −0.19]), lipids (total cholesterol −0.19 mmol/l [−0.33; −0.05], LDL-C mmol/l −0.16 [−0.26; −0.06], HDL-C 0.32 mmol/l [0.02; 0.61]), self-management skill development, and adherence to medication.

**Conclusion:** Pharmacist-led self-management interventions significantly improve HbA1c values in diabetes patients. These results underline the added value of pharmacists in patient-related care. Pharmacists should offer self-management support to diabetes patients in order to improve diabetes outcomes.

## Introduction

Diabetes is a disease which is complex to manage. Treatment consists of lifestyle adaptations often combined with medication to control blood glucose levels (World Health Organization, 2016). Despite available treatment, diabetes is often associated with complications and co-morbidities which increases the complexity of disease management even further (Struijs et al., 2006; Luijks et al., 2012; Lin et al., 2015). Self-management is an essential part of diabetes disease management and is mainly the patient's responsibility. Self-management of chronic conditions has been defined as: “The individual's ability to manage the symptoms, treatment, physical, and psychosocial consequences and life style changes inherent in living with a chronic condition. Efficacious self-management encompasses the ability to monitor one's condition and to effect the cognitive, behavioral, and emotional responses necessary to maintain a satisfactory quality of life.” (Barlow, 2001; Barlow et al., 2002) Patients—especially those with complex diseases—often need support in developing and maintaining self-management skills (Bodenheimer et al., 2002).

Self-management interventions led by physicians, nurses, dieticians, and diabetes educators have been shown to improve HbA1c values in diabetes patients (Newman et al., 2004; Sherifali et al., 2015). Over the years, several reviews have shown that pharmacists also contribute additional value in diabetes care for patients (Machado et al., 2007; Capoccia et al., 2016; Greer et al., 2016; Pousinho et al., 2016). Although, these reviews either studied any type of pharmacist intervention instead of only self-care related interventions (Machado et al., 2007; Greer et al., 2016; Pousinho et al., 2016) or merely focused on adherence (Capoccia et al., 2016). For the U.S., meta-analyses for HbA1c changes were presented by Greer et al. (2016). Machado et al. (2007) presented these figures for studies conducted worldwide. But both studies did not focus on the interventions to improve self-management skills. Furthermore, the meta-analyses either were limited in their scope to only the U.S. or are rather outdated. A comprehensive updated review is needed to summarize the current evidence on the role of pharmacists in supporting self-management skills in diabetes patients. This is all the more important because of the still ongoing paradigm shift of the role of the pharmacist from being a drug supplier to a drug therapy manager (Hepler and Strand, 1990; Wiedenmayer et al., 2006). The aim of this systematic review is to examine the effectiveness of pharmacist-led interventions to support self-management in order to improve clinical- and patient-reported diabetes outcomes.

## Methods

This review was reported according to the PRISMA statement (Moher et al., 2009). The protocol was registered in the Prospero International Prospective Register of Systematic Reviews (registration number: CRD42016041859).

### Research question

This review assessed the effect of pharmacist-led self-management interventions for diabetes patients on clinical- and patient reported outcomes in randomized controlled trials. Primary outcomes were HbA1c, self-management skills, and intervention components. Secondary outcomes were blood glucose, blood pressure, BMI, lipids, adherence to medication, quality of life, and diabetes knowledge.

### Data sources and searches

Pubmed, Embase, Cinahl, PsycINFO, Web of Science, and the Cochrane Library were searched from the start date of the database through to September 2017. Keywords used included “pharmacist,” “diabetes,” and “self-management” (Supplementary Table 1). Whenever possible MeSH terms and advanced searched strategies were used (Supplementary Figure 1). The electronic database searches were complemented by manually reviewing the references of relevant reviews and included studies.

### Study selection

#### Inclusion criteria

A study was included in the review if; (1) the study population was diagnosed with diabetes excluding gestational diabetes, (2) the intervention targeted patients' self-management (Barlow, 2001; Barlow et al., 2002) in an ambulatory care setting, (3) the pharmacist, or a member of the pharmacy team, was involved in the intervention, (4) data on one or more outcome measures were reported e.g., HbA1c, diabetes self-care activities, adherence, (5) the study design was a randomized controlled trial, (6) the full text article was published in either English or Dutch, and (7) it was an original study published in a peer-reviewed journal.

Self-management interventions are not always described as such. Therefore, both direct and indirect self-management interventions were included. By indirect self-management interventions we mean interventions containing components that eventually could lead to improved self-management skills, e.g., diabetes and lifestyle education or concordant goal setting.

#### Study selection

Two reviewers, LvE and LvD, independently assessed all titles and abstracts identified with the initial searches. For all potentially eligible studies the full text papers were obtained via the University of Groningen catalogs, open sources and by emailing first authors. Full text papers were read by both reviewers (LvE and LvD) independently for final inclusion. Any disagreements between the reviewers were resolved by discussion or consultation with a third party (HdG or KT).

### Data extraction and quality assessment

The following data were extracted from the included studies: general study characteristics, description of the study population, follow-up time, number and duration of contact moments during intervention, description, and components of the intervention [diabetes education, medication, lifestyle, individual care plan or goal setting, self-management skills, self-monitoring blood glucose (SMBG) and other, group or individual intervention, education for intervention team], clinical outcomes (HbA1c, blood glucose, blood pressure, BMI, lipid profile, and other), and patient-reported outcomes (adherence, diabetes knowledge, quality of life, self-care/self-management, and other) (Supplementary Table 2). Also it was noted whether interventions were tailored according to the patient's needs. A study was categorized as being tailored if the author made this statement in the research paper. The review team did not base the classification of tailoring on literature statements (Kreuter and Wray, 2003; Noar et al., 2007). The study data were extracted by LvE and double checked for eight papers by LvD, KT, and HdG. Any disagreements were discussed until consensus was reached.

The risk of bias in individual studies was assessed with the Cochrane Risk of Bias tool by LvE (Higgins and Green, 2011). This assessment was double checked by LvD, KT, and HdG by assessing the risk of bias in eight studies. Any disagreements were discussed until consensus was reached.

### Data synthesis and analysis

Interventions across the included studies were analyzed and described narratively.

Outcomes were divided into clinical outcomes (HbA1c, glucose levels, blood pressure, BMI, lipids, and other) and patient-reported outcomes (adherence, diabetes knowledge, quality of life, self-care, and other). Results for HbA1c, blood glucose, blood pressure, BMI, lipids, and Summary of Diabetes Self-care Activities Assessment (SDSCA) were pooled in a meta-analysis. Meta-analyses were performed with Review Manager 5.3 by using a random effects model because of clinical heterogeneity across the included studies. Subgroup analyses were performed for the outcome HbA1c, for different intervention elements (follow-up time, baseline HbA1c ≤ 7% and education for intervention team) in order to explain any heterogeneity (I2) across the studies and to explore key intervention components. Sensitivity analyses were performed to test for robustness of the results regarding including studies with a cluster randomization design and studies with a high risk of bias affecting the outcome HbA1c. Results for adherence, diabetes knowledge and quality of life were described narratively.

## Results

**Figure 1:**
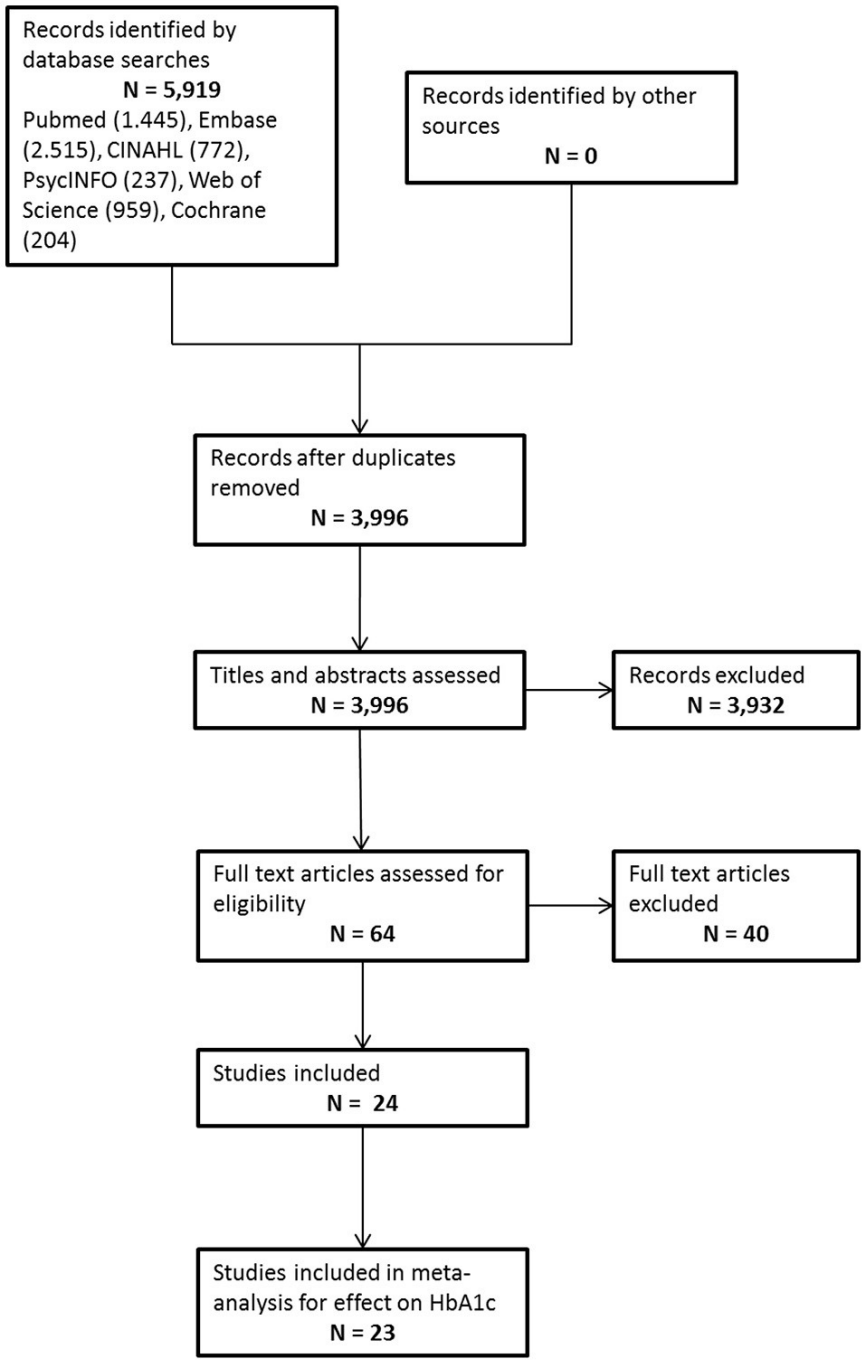
Flow chart study selection.

In total 5,919 hits were identified from the electronic database searches, of which 3,996 were unique. After the title and abstract assessment 3,932 references were excluded because they did not meet the inclusion criteria. The full text of 64 papers was assessed, with 24 papers finally being included in the review. (Figure 1, Supplementary Table 3 for extended data extraction information). Reasons for exclusion after full-text assessment are presented in Supplementary Table 4. Study characteristics of the included studies are presented in Table 1 and characteristics of the study populations of the included studies are presented in Table 2.

**Table 1:** Main study characteristics included studies.

**Author, year, country, study design**	**Follow-up time (in months)**	**Number of contact moments (time)**	**Intervention topics**							**Outcomes**										
											**Clinical**					**Patient reported**				
			**Diabetes education**	**Medication**	**Lifestyle**	**Individual care plan/ goal setting**	**Self-management skills**	**SMBG**	**Other**	**HbA1c**	**Glucose-levels**	**Blood pressure**	**BMI**	**Lipid profile**	**Other**	**Adherence**	**Diabetes knowledge**	**QoL**	**Self-care/ self-management**	**Other**
Armour et al., 2004, Australia, cluster RCT	9	at least 4 visits (NR)		X	X	X	X	X	X	X								X		X
Butt et al., 2015, Malaysia, parallel RCT	6	3 visits (in total 55–75 min.)	X	X	X			X	X	X	X		X	X		X	X	X		
Cani et al., 2015, Brazil, parallel RCT	6	6 visits (NR)	X	X	X			X	X	X						X		X		X
Choe et al., 2005, U.S., parallel RCT	12-24	12 visits/ telephone calls (first visit 60 min.)		X			X		X	X					X					
Cohen et al., 2011, U.S., parallel RCT	6	4 weekly visits (120 min.) + 5 monthly visits (90 min.)	X	X	X	X	X	X	X	X		X		X				X	X	X
Doucette et al., 2009, U.S., parallel RCT	12	4 visits (NR)		X		X	X		X	X		X		X					X	
Farsaei et al., 2011, Iran, parallel RCT	3	2 education sessions followed by weekly phone calls (NR)		X	X	X	X		X	X	X									
Jacobs et al., 2012, U.S., parallel RCT	12	At least 3 visits (NR)	X	X	X	X	X		X	X		X		X						
Jahangard-Rafsanjani et al., 2015, Iran, parallel RCT	5	5 visits (30 min.)	X		X	X	X	X	X	X		X	X			X			X	X
Jameson and Baty, 2010, U.S., parallel RCT	12	On average 6 visits (30–60 min.) + 3 telephone calls (10–20 min.)		X	X		X	X		X										X
Jarab et al., 2012, Jordan, parallel RCT	6	1 visits (NR) + 8 telephone calls (20 min.)	X	X	X			X	X	X	X	X	X	X		X			X	
Kjeldsen et al., 2015, Denmark, parallel RCT	6	At least 4 visits (in total 65–130 min.)		X		X			X			X				X	X	X	X	X
Korcegez et al., 2017, Cyprus, parallel RCT	12	5 visits (NR)	X	X	X	X	X	X	X	X	X	X	X	X		X			X	
Kraemer et al., 2012, U.S., parallel RCT	12	On average 5.4 [4.6; 6.3] visits (NR)				X	X	X		X	X	X		X		X	X		X	
Krass et al., 2007, Australia, cluster RCT	6	5 visits (NR)	X	X	X	X	X	X	X	X		X	X	X				X		
Mehuys et al., 2011, Belgium, cluster RCT	6	visit at start and at each prescription-refill visit (NR)	X	X	X					X	X					X	X		X	
Nascimento et al., 2015, Portugal, parallel RCT	6	at least 2 visits (NR)		X			X			X	X					X			X	
Odegard et al., 2005, U.S., parallel RCT	12	On average 2.1 ± 1.0 visits (30 min.) + 4.5 ± 1.9 telephone calls (10 min.)		X	X	X	X	X	X	X						X				X
Samtia et al., 2013, Pakistan, parallel RCT	5	at least 2 visits (NR)	X	X	X		X	X	X	X	X		X			X	X			X
Sarkadi and Rosenqvist, 2004, Sweden, parallel RCT	12–24	12 visits (NR)	X	X	X		X	X	X	X										X
Shao et al., 2017, China, parallel RCT	6	2 education sessions (NR), 3 face-to-face interviews (NR), 6 telephone interviews (NR)	X	X	X		X	X		X	X	X	X	X		X				
Taveira et al., 2010, U.S., parallel RCT	4	4 weekly group visits (120 min.)	X		X	X	X			X		X	X	X					X	X
Taveira et al., 2011, U.S., parallel RCT	6	4 weekly visits (120 min.) + 4 monthly visits (NR)	X	X	X	X	X	X		X		X		X					X	X
Wishah et al., 2015, Jordan, parallel RCT	6	3 visits (30 min.)	X	X	X	X	X		X	X	X		X	X		X	X		X	

*SMBG, self-monitoring blood glucose; QoL, quality of life*.

**Table 2:** Main characteristics of the study populations.

**Study**		***N***	**Sex (% male)**	**Age (years) (mean, SD)**	**Baseline HbA1c (%, SD)**	**Insulin users (%)**	**DM Type**	**Comorbidities**
Armour et al., 2004	IG	53	45	64 ± 9	7.9 ± 1.5	NR	2	Heart disease, hypertension, hyperlipidemia
	CG	46	51	65 ± 10	7.4 ± 1.2	NR		
Butt et al., 2015	IG	33	39.4	57.4 ± 7.2	9.66 ± 1.57	62.5	2	NR
	CG	33	42.4	57.1 ± 10.8	9.64 ± 1.41	46.3		
Cani et al., 2015	IG	34	38.2	61.9 ± 9.6	9.78 ± 1.55	100	2	NR
	CG	36	38.9	61.6 ± 8.1	9.61 ± 1.38	100		
Choe et al., 2005	IG	41	48.8	52.2 ± 11.2	10.1 ± 1.8	29.3	2	NR
	CG	39	46.1	51.0 ± 9.0	10.2 ± 1.7	30.8		
Cohen et al., 2011	IG	50	100	69.8 ± 10.7	7.8 ± 1.0	NR	2	Heart failure, stroke, coronary heart disease, COPD, mood disorder
	CG	49	96	67.2 ± 9.4	8.1 ± 1.4	NR		
Doucette et al., 2009	IG	31	41.7	58.7 ± 13.3	7.99 ± 1.45	NR	2	NR
	CG	35	47.6	61.2 ± 10.9	7.91 ± 1.91	NR		
Farsaei et al., 2011	IG	87	36.8	53.4 ± 9.8	9.3 ± 1.7	13.1	2	Hypertension, dyslipidemia, heart disease, thyroid disease, renal disease
	CG	87	31.8	52.9 ± 8.5	8.9 ± 1.1	11.5		
Jacobs et al., 2012	IG	72	68	62.7 ± 10.8	9.5 ± 1.1	19	2	Retinopathy, nephropathy, neuropathy
	CG	92	55	63.0 ± 11.2	9.2 ± 1.0	15		
Jahangard-Rafsanjani et al., 2015	IG	45	51	57.3 ± 8.6	7.6 ± 1.6	NR	2	NR
	CG	40	48	55.9 ± 8.7	7.51 ± 1.8	NR		
Jameson and Baty, 2010	IG	52	48.9	49.3 ± 10.8	10.4 ± 1.2	23	NR	NR
	CG	51	49	49.7 ± 10.9	11.1 ± 1.6	28		
Jarab et al., 2012	IG	77	57.6	63.4 ± 10.1	8.5	65.9	2	NR
	CG	79	55.8	65.3 ± 9.2	8.4	69.8		
Kjeldsen et al., 2015	IG-B	33	57.9	63 ± 8.8	NR	NR	2	NR
	IG-E	37	59.5	63.4 ± 7.8	NR	NR		
	CG	102	62.4	62.1 ± 10.2	NR	NR		
Korcegez et al., 2017	IG	75	22.7	61.8 ± 10.38	8.29 ± 0.89	54.7	2	Hypertension, dyslipidemia, thyroid disease, rheumatoid arthritis, asthma, heart failure, osteoporosis, psychological disorders
	CG	77	26.0	62.2 ± 9.54	8.31 ± 0.84	51.9		
Kraemer et al., 2012	IG	36	61.1	55.6 ± 6.8	7.28	13.9	1 & 2	NR
	CG	29	38.7	52.6 ± 9.2	7.38	32.3		
Krass et al., 2007	IG	125	51	62 ± 11	8.9 ± 1.4	NR	2	Hypertension, hyperlipidemia
	CG	107	51	62 ± 11	8.3 ± 1.3	NR		
Mehuys et al., 2011	IG	153	51.0	63	7.7	6.8	2	NR
	CG	135	53.7	62.3	7.3	11.4		
Nascimento et al., 2015	IG	44	56.8	74.2 ± 5.4	8.6 ± 1.2	27.3	2	Hypertension, dyslipidemia, vascular complications
	CG	43	58.1	72.3 ± 4.5	8.2 ± 0.7	34.9		
Odegard et al., 2005	IG	39	52	51.6 ± 11.6	10.2 ± 0.8	26	2	NR
	CG	27	64	51.9 ± 10.4	10.6 ± 1.4	38		
Samtia et al., 2013	IG	108	52.8	46.1	8.51	8.3	2	NR
	CG	97	48.2	42.3	8.54	14.1		
Sarkadi and Rosenqvist, 2004	IG	33	NR	66.4	6.45	NR	2	NR
	CG	31	NR	66.5	6.45	NR		
Shao et al., 2017	IG	99	51.0	58.7 ± 10.59	7.38 ± 1.71	NR	2	NR
	CG	100	47.5	59.2 ± 10.34	7.37 ± 1.44	NR		
Taveira et al., 2010	IG	58	91.4	62.2 ± 10.3	8.5 ± 1.5	NR	2	Hypertension, hyperlipidemia, coronary artery disease, congestive heart failure, COPD
	CG	51	100	66.8 ± 10.2	7.9 ± 1.1	NR		
Taveira et al., 2011	IG	44	100	60.2 ± 9.3	8.3 ± 1.7	NR	2	Depression, coronary artery disease, anxiety, schizophrenia, bipolar, PTSD
	CG	44	95.5	61.4 ± 9.9	8.5 ± 1.9	NR		
Wishah et al., 2015	IG	52	38.5	52.9 ± 9.6	8.9 ± 1.6	NR	2	NR
	CG	54	48.1	53.2 ± 11.2	8.2 ± 1.3	NR		

*CG, control group; IG, intervention group; IG-B, basic intervention group; IG-E, extended intervention group*.

### Description of included studies

Three of the included studies had a cluster randomized design (Armour et al., 2004; Krass et al., 2007; Mehuys et al., 2011) and 21 were randomized controlled trials (Sarkadi and Rosenqvist, 2004; Choe et al., 2005; Odegard et al., 2005; Doucette et al., 2009; Jameson and Baty, 2010; Taveira et al., 2010, 2011; Cohen et al., 2011; Farsaei et al., 2011; Jacobs et al., 2012; Jarab et al., 2012; Kraemer et al., 2012; Samtia et al., 2013; Butt et al., 2015; Cani et al., 2015; Jahangard-Rafsanjani et al., 2015; Kjeldsen et al., 2015; Nascimento et al., 2015; Wishah et al., 2015; Korcegez et al., 2017; Shao et al., 2017) (Table 1). All studies were published from 2004 onwards. Most of the studies were conducted in North America (Choe et al., 2005; Odegard et al., 2005; Doucette et al., 2009; Jameson and Baty, 2010; Taveira et al., 2010, 2011; Cohen et al., 2011; Jacobs et al., 2012; Kraemer et al., 2012) (9), followed by Asia (Farsaei et al., 2011; Jarab et al., 2012; Samtia et al., 2013; Butt et al., 2015; Jahangard-Rafsanjani et al., 2015; Wishah et al., 2015; Shao et al., 2017) (7), Europe (Sarkadi and Rosenqvist, 2004; Mehuys et al., 2011; Kjeldsen et al., 2015; Nascimento et al., 2015; Korcegez et al., 2017) (5), Australia (Armour et al., 2004; Krass et al., 2007) (2), and South America (Cani et al., 2015) (1). The majority of the studies focused primarily on diabetes mellitus type 2 patients (Armour et al., 2004; Sarkadi and Rosenqvist, 2004; Choe et al., 2005; Odegard et al., 2005; Krass et al., 2007; Doucette et al., 2009; Taveira et al., 2010, 2011; Cohen et al., 2011; Farsaei et al., 2011; Mehuys et al., 2011; Jacobs et al., 2012; Jarab et al., 2012; Samtia et al., 2013; Butt et al., 2015; Cani et al., 2015; Jahangard-Rafsanjani et al., 2015; Kjeldsen et al., 2015; Nascimento et al., 2015; Wishah et al., 2015; Korcegez et al., 2017; Shao et al., 2017) (22), one study included both type 1 and type 2 patients (Kraemer et al., 2012) and one study did not specify the type of diabetes (Jameson and Baty, 2010). In total the included studies represented 3,610 participants with a mean age ranging from 44 to 73 years of age. The median follow-up time was 6 months (Krass et al., 2007; Cohen et al., 2011; Mehuys et al., 2011; Taveira et al., 2011; Jarab et al., 2012; Butt et al., 2015; Cani et al., 2015; Kjeldsen et al., 2015; Nascimento et al., 2015; Wishah et al., 2015; Korcegez et al., 2017), four studies had a follow-up time of less than 6 months (Taveira et al., 2010; Farsaei et al., 2011; Samtia et al., 2013; Jahangard-Rafsanjani et al., 2015) and 10 of more than 6 months (Armour et al., 2004; Sarkadi and Rosenqvist, 2004; Choe et al., 2005; Odegard et al., 2005; Doucette et al., 2009; Jameson and Baty, 2010; Jacobs et al., 2012; Kraemer et al., 2012; Shao et al., 2017).

### Description of intervention

The interventions in the included studies were all provided by a trained pharmacist, either by the pharmacist alone (Armour et al., 2004; Choe et al., 2005; Odegard et al., 2005; Krass et al., 2007; Doucette et al., 2009; Jameson and Baty, 2010; Farsaei et al., 2011; Mehuys et al., 2011; Jacobs et al., 2012; Jarab et al., 2012; Kraemer et al., 2012; Samtia et al., 2013; Butt et al., 2015; Cani et al., 2015; Jahangard-Rafsanjani et al., 2015; Kjeldsen et al., 2015; Wishah et al., 2015; Korcegez et al., 2017; Shao et al., 2017) or within a multi-disciplinary team (Sarkadi and Rosenqvist, 2004; Taveira et al., 2010, 2011; Cohen et al., 2011). One study did not specify the intervention team, besides including a pharmacist (Nascimento et al., 2015). Most interventions targeted the individual patient (Armour et al., 2004; Choe et al., 2005; Odegard et al., 2005; Krass et al., 2007; Doucette et al., 2009; Jameson and Baty, 2010; Mehuys et al., 2011; Jacobs et al., 2012; Jarab et al., 2012; Kraemer et al., 2012; Samtia et al., 2013; Butt et al., 2015; Cani et al., 2015; Jahangard-Rafsanjani et al., 2015; Kjeldsen et al., 2015; Wishah et al., 2015; Korcegez et al., 2017; Shao et al., 2017) whereas some interventions used group sessions (Sarkadi and Rosenqvist, 2004; Taveira et al., 2010, 2011; Cohen et al., 2011). One study did not specify whether the intervention was offered in an individual or group setting (Farsaei et al., 2011). Fifteen studies reported offering a tailored intervention based on a patient's specific needs (Armour et al., 2004; Odegard et al., 2005; Krass et al., 2007; Jameson and Baty, 2010; Taveira et al., 2010, 2011; Farsaei et al., 2011; Jarab et al., 2012; Kraemer et al., 2012; Cani et al., 2015; Jahangard-Rafsanjani et al., 2015; Kjeldsen et al., 2015; Nascimento et al., 2015; Wishah et al., 2015; Korcegez et al., 2017).

The interventions in the included studies varied in the intensity as well as the number and type of components. The intensity, measured as the frequency of contact moments, differed across the studies from once a week to once every 3 months. Face-to-face contact with the pharmacists (Armour et al., 2004; Sarkadi and Rosenqvist, 2004; Krass et al., 2007; Doucette et al., 2009; Taveira et al., 2010, 2011; Cohen et al., 2011; Mehuys et al., 2011; Jacobs et al., 2012; Kraemer et al., 2012; Samtia et al., 2013; Butt et al., 2015; Cani et al., 2015; Jahangard-Rafsanjani et al., 2015; Kjeldsen et al., 2015; Nascimento et al., 2015; Wishah et al., 2015; Korcegez et al., 2017) (18) as well as a combination of face-to-face contacts and telephone contact with the pharmacists (Choe et al., 2005; Odegard et al., 2005; Jameson and Baty, 2010; Farsaei et al., 2011; Jarab et al., 2012; Shao et al., 2017) (6) were reported in the studies. The total contact time varied across the studies, though not all studies reported this information (Armour et al., 2004; Sarkadi and Rosenqvist, 2004; Doucette et al., 2009; Farsaei et al., 2011; Mehuys et al., 2011; Taveira et al., 2011; Jacobs et al., 2012; Samtia et al., 2013; Cani et al., 2015; Nascimento et al., 2015; Korcegez et al., 2017; Shao et al., 2017). (Table 1). Fifteen studies included diabetes education (Sarkadi and Rosenqvist, 2004; Krass et al., 2007; Taveira et al., 2010, 2011; Cohen et al., 2011; Mehuys et al., 2011; Jacobs et al., 2012; Jarab et al., 2012; Samtia et al., 2013; Butt et al., 2015; Cani et al., 2015; Jahangard-Rafsanjani et al., 2015; Wishah et al., 2015; Korcegez et al., 2017; Shao et al., 2017) either about diabetes in general or about acute and chronic complications. Education on medication was provided in 21 studies (Armour et al., 2004; Sarkadi and Rosenqvist, 2004; Choe et al., 2005; Odegard et al., 2005; Krass et al., 2007; Doucette et al., 2009; Jameson and Baty, 2010; Cohen et al., 2011; Farsaei et al., 2011; Mehuys et al., 2011; Taveira et al., 2011; Jacobs et al., 2012; Jarab et al., 2012; Samtia et al., 2013; Butt et al., 2015; Cani et al., 2015; Kjeldsen et al., 2015; Nascimento et al., 2015; Wishah et al., 2015; Korcegez et al., 2017; Shao et al., 2017) and included education about adherence, dosage, drug-related problems, indication, storage, and use. In 19 studies education on lifestyle, including diet, exercise, foot care, and/or smoking cessation were part of the intervention (Armour et al., 2004; Sarkadi and Rosenqvist, 2004; Odegard et al., 2005; Krass et al., 2007; Jameson and Baty, 2010; Taveira et al., 2010, 2011; Cohen et al., 2011; Farsaei et al., 2011; Mehuys et al., 2011; Jacobs et al., 2012; Jarab et al., 2012; Samtia et al., 2013; Butt et al., 2015; Cani et al., 2015; Jahangard-Rafsanjani et al., 2015; Wishah et al., 2015; Korcegez et al., 2017; Shao et al., 2017). In 19 studies the intervention included self-management skills support (Armour et al., 2004; Sarkadi and Rosenqvist, 2004; Choe et al., 2005; Odegard et al., 2005; Krass et al., 2007; Doucette et al., 2009; Jameson and Baty, 2010; Taveira et al., 2010, 2011; Cohen et al., 2011; Farsaei et al., 2011; Jacobs et al., 2012; Kraemer et al., 2012; Samtia et al., 2013; Jahangard-Rafsanjani et al., 2015; Nascimento et al., 2015; Wishah et al., 2015; Korcegez et al., 2017; Shao et al., 2017) and in 15 studies participants were trained in self-monitoring blood glucose (Armour et al., 2004; Sarkadi and Rosenqvist, 2004; Odegard et al., 2005; Krass et al., 2007; Jameson and Baty, 2010; Cohen et al., 2011; Taveira et al., 2011; Jarab et al., 2012; Kraemer et al., 2012; Samtia et al., 2013; Butt et al., 2015; Cani et al., 2015; Jahangard-Rafsanjani et al., 2015; Korcegez et al., 2017; Shao et al., 2017). A total of 14 studies used either an individual care plan or goal setting to improve diabetes outcomes (Armour et al., 2004; Odegard et al., 2005; Krass et al., 2007; Doucette et al., 2009; Taveira et al., 2010, 2011; Cohen et al., 2011; Farsaei et al., 2011; Jacobs et al., 2012; Kraemer et al., 2012; Jahangard-Rafsanjani et al., 2015; Kjeldsen et al., 2015; Wishah et al., 2015; Korcegez et al., 2017). Other less common interventions were the use of a diabetes diary (Farsaei et al., 2011; Butt et al., 2015; Jahangard-Rafsanjani et al., 2015), medication reviews by a pharmacist (Armour et al., 2004; Choe et al., 2005; Krass et al., 2007; Doucette et al., 2009; Jacobs et al., 2012; Korcegez et al., 2017), and providing participants with written information (Sarkadi and Rosenqvist, 2004; Jarab et al., 2012; Cani et al., 2015; Jahangard-Rafsanjani et al., 2015; Nascimento et al., 2015; Wishah et al., 2015; Korcegez et al., 2017).

Many different outcome measures were reported by the included studies (Table 1). They were divided in clinical and patient-reported outcomes.

### Clinical outcomes

All studies reported HbA1c as an outcome measurement for their intervention. A meta-analysis was performed, with one study excluded because of an insufficient number of participants reporting HbA1c at the final follow-up (Kjeldsen et al., 2015).

**Figure 2:**
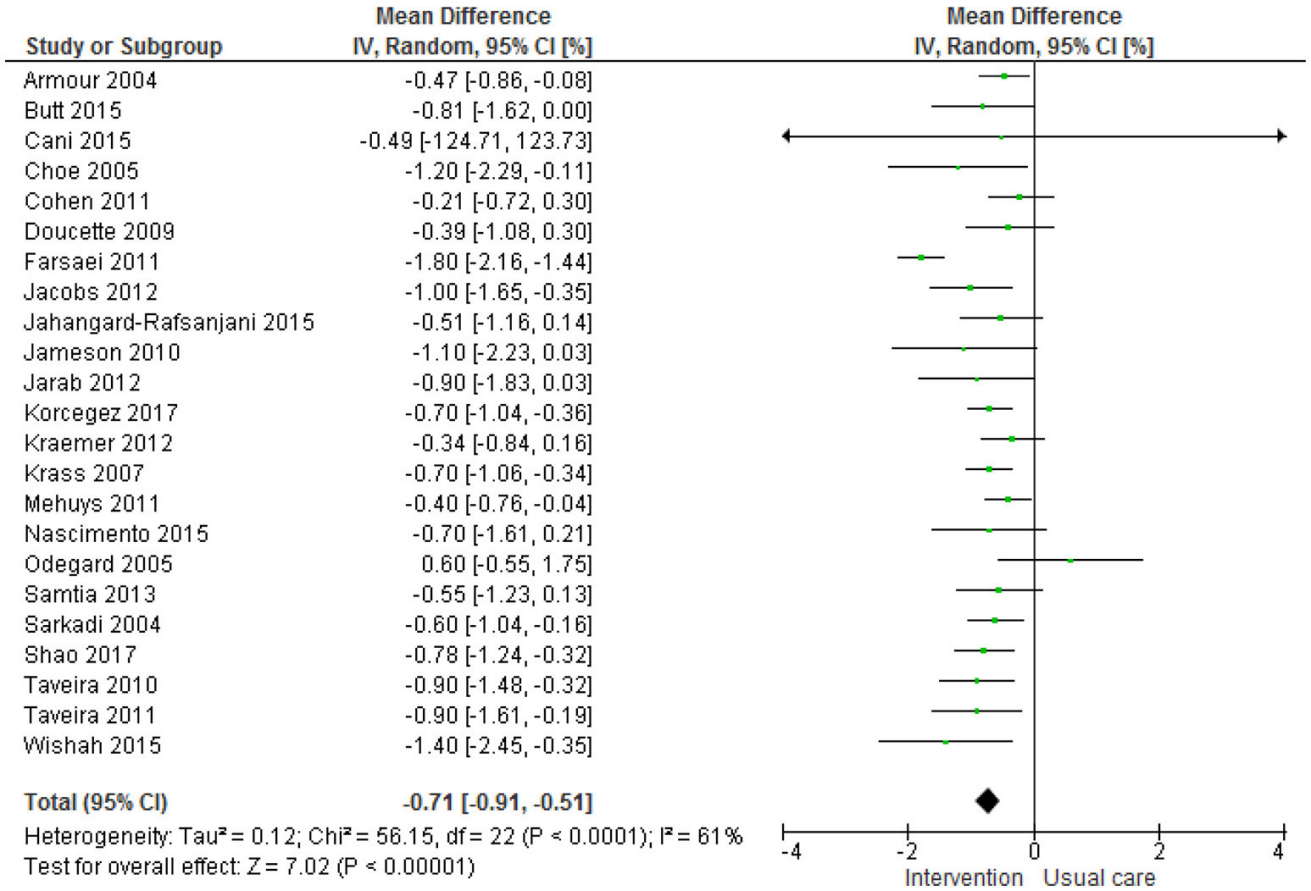
Meta-analysis HbA1c.

The meta-analysis (Figure 2) shows an overall significant effect in favor of the intervention on HbA1c, with HbA1c levels improving by a mean of 0.71% (CI −0.91, −0.51; overall effect *P* < 0.0001). Several subgroup analyses were performed based on different study characteristics (Table 3, Supplementary Figures 2A–I). None of these subgroup analyses showed a significant difference between groups.

**Table 3:** Subgroup analyses HbA1c.

	**Subgroup**	***N***	**I2 (*P*-value)**	**CI**	**Test for subgroup differences**
1	Overall	23	61% (*P* < 0.0001)	−0.71 [−0.91; −0.51]	–
2.1	Tailored intervention				*P* = 0.33, I2 = 0%
2.1.1	Yes	13	72% (*P* < 0.0001)	−0.79 [−1.14; −0.44]	
2.1.2	No	10	0% (*P* = 0.58)	−0.60 [−0.76; −0.44]	
3.1	Group vs. individual intervention				*P* = 0.53; I2 = 0%
3.1.1	Group	4	23% (*P* = 0.27)	−0.61 [−0.92; −0.30]	
3.1.2	Individual	19	65% (*P* < 0.0001)	−0.73 [−0.97; −0.50]	
4.1	Follow-up time				*P* = 0.57; I2 = 0%
4.1.1	< 6 months	4	84% (*P* = 0.0002)	−0.98 [−1.68; −0.28]	
4.1.2	6 months	10	0% (*P* = 0.55)	−0.62 [−0.80; −0.44]	
4.1.3	>6 months	9	17% (*P* = 0.29)	−0.58 [−0.79; −0.37]	
5.1	Follow-up time				*P* = 0.30; I2 = 5.0%
5.1.1	< 6 months	4	84% (*P* = 0.0002)	−0.98 [−1.68; −0.28]	
5.1.2	≥6 months	19	0% (*P* = 0.48)	−0.60 [−0.73; −0.48]	
6.1	HbA1c baseline cut off 7%				*P* = 0.21; I2 = 37.7%
6.1.1	< 7%	2	0% (*P* = 0.45)	−0.49 [−0.82; −0.15]	
6.1.2	>7%	21	62% (*P* < 0.0001)	−0.74[−0.96; −0.52]	
7.1	Education intervention team				*P* = 0.05; I2 = 75%
7.1.1	Yes	7	0% (*P* = 0.90)	−0.51 [−0.68; −0.34]	
7.1.2	No	16	64% (*P* = 0.0003)	−0.85 [−1.14; −0.56]	
8.1	Adherence				*P* = 0.35; I2 = 0%
8.1.1	Yes	12	76% (*P* < 0.00001)	−0.77 [−1.09; −0.46]	
8.1.2	No	11	0% (*P* = 0.63)	−0.60 [−0.79; −0.40]	
9.1	DRP/side effects				*P* = 0.26; I2 = 20.1%
9.1.1	Yes	9	79% (*P* < 0.00001)	−0.84 [−1.24; −0.41]	
9.1.2	No	14	0% (*P* = 0.68)	−0.57 [−0.73; −0.41]	
10.1	Individual Care Plan/Goal setting				*P* = 0.42; I2 = 0%
10.1.1	Yes	11	77% (*P* < 0.00001)	−0.76 [−1.11; −0.40]	
10.1.2	No	12	0% (*P* = 0.67)	−0.60 [−0.77; −0.42]	

Other clinical outcomes reported were blood glucose levels, blood pressure, BMI, and lipid profile (Table 4, Supplementary Figures 3–6). Meta-analyses showed no significant reduction for blood glucose levels, but a significant improvement in systolic- and diastolic blood pressure (−5.20 mm Hg [−7.58; −2.92] and −3.51 mm Hg [−6.00; −1.01], respectively), BMI scores (−0.49 kg/m2 [−0.79; −0.19]), total cholesterol levels (−0.19 mmol/l [−0.33; −0.05]), LDL-C levels (−0.16 mmol/l [−0.26; −0.06]), and HDL-C levels (0.32 mmol/l [0.02; 0.61]).

**Table 4:** Pooled outcomes clinical parameters.

**Outcome**	**Pooled results (mean, CI)**
Blood glucose (mmol/l) (Farsaei et al., 2011; Mehuys et al., 2011; Jarab et al., 2012; Kraemer et al., 2012; Samtia et al., 2013; Butt et al., 2015; Nascimento et al., 2015; Wishah et al., 2015; Korcegez et al., 2017; Shao et al., 2017)	−0.26 [−0.97; 0.46]
Blood pressure (mm Hg)	
Systolic blood pressure (Krass et al., 2007; Doucette et al., 2009; Taveira et al., 2010, 2011; Cohen et al., 2011; Jacobs et al., 2012; Jarab et al., 2012; Kraemer et al., 2012; Jahangard-Rafsanjani et al., 2015; Korcegez et al., 2017; Shao et al., 2017)	−5.20 [−7.48; −2.92]
Diastolic blood pressure (Krass et al., 2007; Doucette et al., 2009; Taveira et al., 2010; Jacobs et al., 2012; Jarab et al., 2012; Kraemer et al., 2012; Jahangard-Rafsanjani et al., 2015; Korcegez et al., 2017; Shao et al., 2017)	−3.51 [−6.00; −1.01]
BMI (kg/m2) (Taveira et al., 2010; Jarab et al., 2012; Samtia et al., 2013; Butt et al., 2015; Jahangard-Rafsanjani et al., 2015; Wishah et al., 2015; Korcegez et al., 2017; Shao et al., 2017)	−0.49 [−0.79; −0.19]
Lipids (mmol/l)	
Total cholesterol (Krass et al., 2007; Jarab et al., 2012; Kraemer et al., 2012; Butt et al., 2015; Wishah et al., 2015; Korcegez et al., 2017; Shao et al., 2017)	−0.19 [−0.33; −0.05]
LDL-C (Doucette et al., 2009; Taveira et al., 2010, 2011; Cohen et al., 2011; Jacobs et al., 2012; Jarab et al., 2012; Kraemer et al., 2012; Butt et al., 2015; Wishah et al., 2015; Korcegez et al., 2017; Shao et al., 2017)	−0.16 [−0.26; −0.06]
HDL-C (Jarab et al., 2012; Kraemer et al., 2012; Butt et al., 2015; Wishah et al., 2015; Korcegez et al., 2017; Shao et al., 2017)	0.32 [0.02; 0.61]
Triglycerides (Krass et al., 2007; Jarab et al., 2012; Kraemer et al., 2012; Butt et al., 2015; Wishah et al., 2015; Korcegez et al., 2017; Shao et al., 2017)	−0.01 [−0.06; 0.03]

### Patient reported outcomes

#### Self-management

**Figure 3:**
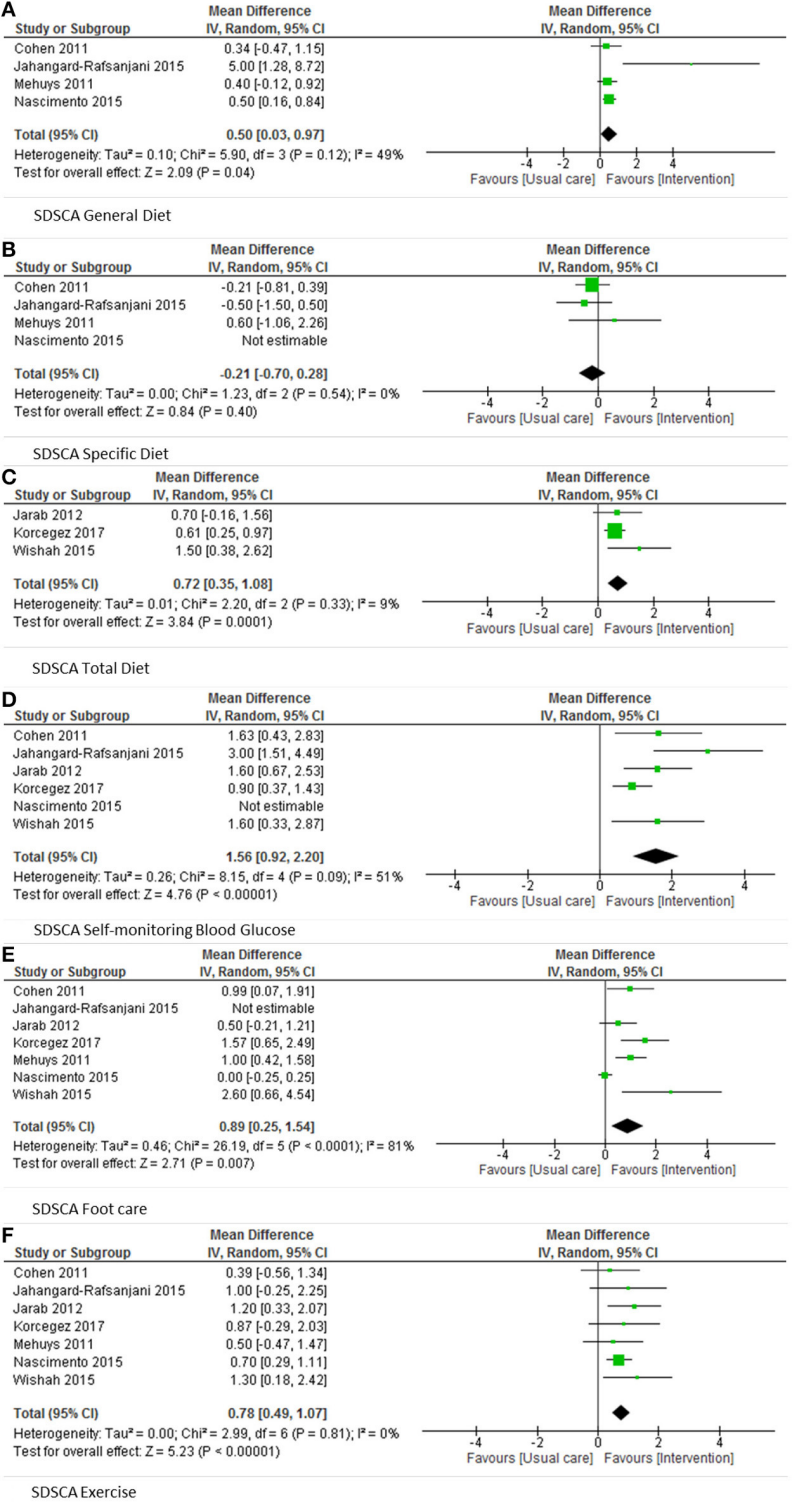
Pooled results SDSCA categories.

Adherence to diabetes self-care was assessed in 12 studies (Doucette et al., 2009; Taveira et al., 2010, 2011; Cohen et al., 2011; Mehuys et al., 2011; Jarab et al., 2012; Kraemer et al., 2012; Jahangard-Rafsanjani et al., 2015; Kjeldsen et al., 2015; Nascimento et al., 2015; Wishah et al., 2015; Korcegez et al., 2017). Nine of them used the validated Summary of Diabetes Self-Care Activities assessment (SDSCA) (Doucette et al., 2009; Cohen et al., 2011; Mehuys et al., 2011; Taveira et al., 2011; Jarab et al., 2012; Jahangard-Rafsanjani et al., 2015; Nascimento et al., 2015; Wishah et al., 2015; Korcegez et al., 2017). This questionnaire consists of five domains (general diet, specific diet, exercise, self-monitoring blood glucose, foot care), and domain scores as well as an overall score can be calculated. Six studies reported domain scores (Cohen et al., 2011; Mehuys et al., 2011; Jarab et al., 2012; Jahangard-Rafsanjani et al., 2015; Nascimento et al., 2015; Wishah et al., 2015). The results of these six studies were pooled in a meta-analysis and a significant effect of pharmacist-led interventions was found for general diet, self-monitoring blood glucose, foot care, and exercise (Figures 3A–F).

#### Adherence to medication

Adherence to medication was measured in 13 studies (Odegard et al., 2005; Mehuys et al., 2011; Jarab et al., 2012; Kraemer et al., 2012; Samtia et al., 2013; Butt et al., 2015; Cani et al., 2015; Jahangard-Rafsanjani et al., 2015; Kjeldsen et al., 2015; Nascimento et al., 2015; Wishah et al., 2015; Korcegez et al., 2017; Shao et al., 2017). Seven studies (Jarab et al., 2012; Butt et al., 2015; Cani et al., 2015; Jahangard-Rafsanjani et al., 2015; Wishah et al., 2015; Korcegez et al., 2017; Shao et al., 2017) used the validated Morisky-Green questionnaire. Due to different reporting strategies it was not possible to pool the results. Six studies reported significant improvement in adherence in the intervention group compared to the control group and one study reported improved adherence outcomes within the intervention group but did not compare intervention and control group (Korcegez et al., 2017).

#### Quality of life

Six studies (Armour et al., 2004; Krass et al., 2007; Cohen et al., 2011; Butt et al., 2015; Cani et al., 2015; Kjeldsen et al., 2015) reported quality of life outcomes, of which three studies (Krass et al., 2007; Butt et al., 2015; Kjeldsen et al., 2015) used the validated EQ-5D(-3L) questionnaire. Due to the use of different versions of the questionnaire and differences in reporting strategies it was not possible to pool the results. Two studies reported significantly improved quality of life based on the results from the EQ-5D tool (Krass et al., 2007; Butt et al., 2015).

#### Diabetes knowledge

Diabetes knowledge was reported in six studies (Mehuys et al., 2011; Kraemer et al., 2012; Samtia et al., 2013; Butt et al., 2015; Kjeldsen et al., 2015; Wishah et al., 2015), of which three studies (Mehuys et al., 2011; Kraemer et al., 2012; Wishah et al., 2015) used the validated Diabetes Knowledge Test of The Michigan Diabetes Research and Training Center. Due to the use of different reporting strategies it was not possible to pool the results. Only Wishah et al. (2015) reported significant improvement of diabetes knowledge.

### Risk of bias

The risk of bias within studies was assessed with the Cochrane Risk of Bias tool. All but two (Taveira et al., 2011; Jarab et al., 2012) studies were subjected to some form of bias either at high risk or at an unclear risk due to lack of information (Supplementary Figure 7). In total, eight studies were considered to have a low risk of bias (Sarkadi and Rosenqvist, 2004; Jameson and Baty, 2010; Taveira et al., 2011; Jacobs et al., 2012; Jarab et al., 2012; Butt et al., 2015; Cani et al., 2015; Wishah et al., 2015).

### Publication bias

The funnel plot for the pooled results of HbA1c can be considered symmetric and indicates that it is unlikely publication bias has been introduced in the analysis (Supplementary Figure 8).

### Sensitivity analysis

Two sensitivity analyses were performed. In the first sensitivity analysis the studies with a cluster randomization design were excluded, because none of these studies corrected for the clustering effect. The clustering effect is known for potential overestimation of the effect of the intervention (Killip et al., 2004). After excluding these studies the weighted mean difference of HbA1c for the patient-level randomized studies was −0.76% [−1.00; −0.52]. This difference is of the same magnitude as the difference observed when including all studies.

The second sensitivity analysis was performed using only the eight studies with a relatively low risk of bias from influences on HbA1c. The weighted mean difference of HbA1c for studies with a low risk of bias was −0.84% [−1.11; −0.57]. This difference is also of the same magnitude as the difference observed when including all studies.

## Discussion

### Summary of main findings

This review found evidence that pharmacist-led self-management interventions are beneficial for diabetes patients. All of the included studies used proxies to measure the effect of self-management interventions; only a minority directly measured the effect of self-management interventions on self-management skills. Overall, pharmacist-led interventions had a positive effect on HbA1c values, blood pressure, BMI, and self-management skills as shown by the results of the meta-analyses. Also the results suggest pharmacist-led self-management interventions improve adherence to medication, diabetes knowledge, and quality of life.

The results on HbA1c values in the meta-analysis showed a significant effect of pharmacist-led interventions. The magnitude of this reduction (−0.71% [−0.91; −0.51]) can be considered as clinically relevant and can be associated with risk reduction in microvascular complications (Stratton et al., 2000). These findings are in agreement with the findings of Machado et al. (2007), who reported a pooled effect of −1.00 ± 0.28% on HbA1c values. However, in their review all kinds of pharmacist interventions for diabetes patients were included. Compared to systematic reviews on the effect of self-management interventions by either a physician, nurse or diabetes educator, the effect of pharmacist-led self-management interventions was over three times larger (Sherifali et al., 2015). The added value of pharmacist-led interventions for diabetes goal attainment is supported by the findings of Greer et al. (2016), who reported a relative risk (1.83 [1.44; 2.33]) in favor of diabetes patients receiving pharmacist-led disease management. The diversity of intervention contents in the included studies is also highlighted in previous reviews (Machado et al., 2007; Greer et al., 2016; Pousinho et al., 2016).

### Strengths and limitations

This study has several strengths. All of the studies included measured HbA1c values, which made it possible to compare the effect of the described interventions in a meta-analysis. Also the results for blood glucose, blood pressure, BMI, lipids, and self-management skills could be pooled in meta-analyses.

Though most studies used proxies to measure the effect of pharmacist-led self-management interventions, a few studies directly measured self-management. The results of these studies reveal a positive direct relation between the self-management intervention and the development of self-management skills in diabetes patients. This is most likely because the interventions in almost all of the included studies addressed medication and medication-related problems that are rather common among diabetes patients (Haugbolle and Westh Sorensen, 2006; Kempen et al., 2014).

This study also has some limitations. The reporting of the interventions and study results were very limited in some of the studies (Armour et al., 2004; Choe et al., 2005; Odegard et al., 2005; Krass et al., 2007; Doucette et al., 2009; Taveira et al., 2010; Cohen et al., 2011; Farsaei et al., 2011; Mehuys et al., 2011; Kraemer et al., 2012; Samtia et al., 2013; Jahangard-Rafsanjani et al., 2015; Kjeldsen et al., 2015; Nascimento et al., 2015). This made the risk of bias assessment difficult. However, the sensitivity analysis showed that excluding studies with a high risk of bias did not materially change the results of the meta-analysis of the HbA1c values.

The most frequently used instrument to measure self-management in diabetes patients was the SDSCA questionnaire. However, the SDSCA questionnaire pays limited attention to medication related issues (Toobert et al., 2000). Therefore, this questionnaire may not be the best instrument to measure the effects of pharmacist-led and medication-related self-management support. A more suitable instruments for instance might be the MUSE questionnaire (Medication Understanding and Use Self-Efficacy Scale), which focuses on medication use and knowledge (Cameron et al., 2010). This scale can be used among patients with any level of health literacy.

The interventions reported in all of the included studies can be considered as complex interventions, because all of them consisted of multiple components. Also the mechanisms of action for implicating practice were complex as this depended on both the pharmacists delivering and implementing the intervention and the patient implementing it into daily life (Kelly et al., 2017). In this review we have shown that these complex interventions have a positive influence on various diabetes related outcomes. Subgroup analyses did not provide evidence which of the components were essential for the effect. More sophisticated analyses, such as meta-regression analyses or modeling, could have given more insight into key components (Viswanathan et al., 2017). However, this was not possible due to the limited number of studies, data available and the different ways in which the data was presented in the included studies. Although, we have described the different components of pharmacist-led self-management interventions, the ideal composition of intervention components is still a black box.

### Clinical implications and future research

The overall results of our study argue that pharmacists take an active role in improving patient diabetes self-management since the effectiveness of pharmacist-led interventions is at least comparable to that of other healthcare providers (Sherifali et al., 2015). Although we were unable to identify specific factors contributing to the success of pharmacist-led self-management interventions, a tailored approach seems to be preferable for future developments (Linn et al., 2011, 2013; Harrington and Noar, 2012). In line with findings of previous studies; self-management needs depend on personal characteristics and development (Bos-Touwen et al., 2015) and self-management support should focus on how to identify problems and how to take appropriate actions (Bodenheimer et al., 2002). Another important factor for successful interventions might be the intensity of contact moments over time, with the intensity of contact moments appearing more important than the length of the intervention. This is demonstrated by Krass et al. (2011) and Odegard et al. (2005) who found that prolonging the follow-up time without sustaining the contact frequency did not further improve HbA1c values. Moreover, some patient groups are more vulnerable to having low self-management skills than others. For example, patients with a low level of health literacy may benefit much more from self-management support compared to more health literate diabetes patients (Fransen et al., 2012, 2015). Summarizing the evidence, pharmacists should offer self-management support to diabetes patients in order to improve clinical- and patient reported diabetes outcomes.

Future research into self-management support should focus on developing an intervention from a multidisciplinary perspective to combine the knowledge from the different disciplines involved in diabetes care. Most studies only focus on the role of a single healthcare professional. Combining the strengths of different disciplines might increase the effect of the intervention. Particular emphasis should be placed on vulnerable patient groups and using valid measurements of self-management skills in multiple dimensions.

## Conclusion

This review demonstrates that pharmacists contribute additional value in self-management support interventions for diabetes patients. Pharmacists are involved in a variety of different self-management interventions, which vary in many key aspects such as follow-up time and use of a tailored approach. Overall pharmacist-led self-management interventions have a positive effect on lowering HbA1c values.

## Author contributions

LvE: Main researcher in this review. Involved in all parts of the review including; research protocol, search strategy, literature search, study selection, risk of bias, data extraction, data analysis, and composing the manuscript. LvD: Study selection, risk of bias, data extraction, and revising manuscript. KT: Search strategy, risk of bias, data extraction, revising manuscript. HdG: Protocol, risk of bias, data extraction, revising manuscript.

### Conflict of interest statement

The authors declare that the research was conducted in the absence of any commercial or financial relationships that could be construed as a potential conflict of interest.
