# Supplementary material for: Pharmacist-Led Self-management Interventions to Improve Diabetes Outcomes. A Systematic Literature Review and Meta-Analysis
Source: Front Pharmacol. 2017 Dec 14;8:891. doi: 10.3389/fphar.2017.00891 (PMC5735079; doi:10.3389/fphar.2017.00891)
Supplement: Supplementary file 1 [file DataSheet1.docx]

*Supplementary Material*

**Pharmacist-led self-management interventions to improve diabetes outcomes. *A systematic literature review and meta-analysis.***

**Linda van Eikenhorst*, MSc, Katja Taxis, PhD, Liset van Dijk, PhD, Han de Gier, PhD**

***Correspondence**: Corresponding Author: l.van.eikenhorst@rug.nl

Supplementary Table 1: Keyword Search Terms

Supplementary Table 2: Data Extraction Categories

Supplementary Table 3: Extended data extraction included studies

Supplementary Table 4: Exclusion reasons for full text papers

Supplementary Figure 1: Search strategy PubMed

Supplementary Figure 2a-i: Forest plots subgroup analyses HbA1c

Supplementary Figure 3: Forest plot Blood glucose

Supplementary Figure 4: Forest plot Blood pressure

Supplementary Figure 5: Forest plot BMI

Supplementary Figure 6: Forest plots Lipids

Supplementary Figure 7: Risk of Bias

Supplementary Figure 8: Funnel plot of publication bias

Supplementary Table 1: Keyword Search Terms

|  | Term | Text words |
| --- | --- | --- |
| 1 | Pharmacist | Pharmacist, pharmacists, pharmacy, pharmacies, pharmaceutical |
| 2 | Diabetes | Diabetes, DM, diabetic |
| 3 | Self-management | Self-management, self management, self care, self-care, self efficacy, self-efficacy, patient participation, medication management, adherence, nonadherence, compliance, noncompliance |

Supplementary Table 2: Data Extraction Categories

| Category | Information |
| --- | --- |
| General | First author, year of publication, country study was conducted, study design (parallel RCT or cluster RCT) |
| Study characteristics | Study setting, study design, follow-up period, sample size |
| Study population | Sex, age, baseline HbA1c, comorbidities |
| Intervention | Description of intervention, frequency of meetings, duration of meetings, intervention team, education for the intervention team.  Intervention topics; diabetes education (complications, disease in general), medication (adherence, dosage, drug related problems, indication, insulin technique, side effects, storage, use), lifestyle (diet, exercise, eye examination, foot care, lifestyle, smoking cessation), individual care plan/goal setting, self-management skills, self-monitoring blood glucose and other (diabetes diary, discuss health beliefs, general health, identify problems, medical checks, medication review, monitor blood glucose by pharmacist, physical assessment, rationalize therapy, written information). |
| Outcomes | Clinical outcomes; glucose control (HbA1c, blood glucose), blood pressure (systolic blood pressure, diastolic blood pressure), body measures (BMI, height, weight, waist circumference), lipids (LDL, HDL, triglycerides, total cholesterol), other (dilated retinal examination, eGFR, test for diabetes neuropathy, urine microalbumine screening)  Patient reported outcomes; adherence, diabetes knowledge, quality of life, self-care/self-management, other (BMQ, cardiovascular risk, death, depressive symptoms, glucose monitoring technique, hospital admissions, number of interventions delivered, medication knowledge, patient health questions, patient perceived competence, personal perception of diabetes, number of pharmacy visits, number of physician visits, tobacco use) |

Supplementary Table 3: Extended data extraction included studies

| Author | Armour (Armour et al., 2004) | | |
| --- | --- | --- | --- |
| Year | 2004 | | |
| Country | Australia | | |
| Objective | To develop, implement, and evaluate a disease management service model for type 2 diabetes in community pharmacy. | | |
| Study setting | Outpatient diabetes clinic | | |
| Study design | Cluster randomized controlled trial | | |
| Follow-up period | 9 months | | |
| Sample | N | 239 | |
|  |  | Intervention | Control |
|  | Sex (% male) | 45 | 51 |
|  | Age (years ± SD) | 64 ± 9 | 65 ± 10 |
|  | Baseline HbA1c (% ± SD) | 7.9 ± 1.5 | 7.4 ± 1.2 |
|  | Comorbidities | Heart disease, hypertension, hyperlipidemia | |
| Intervention |  | | |
| Description | All pharmacists conducted a medication review and monitored blood glucose levels of patients. Discretionary interventions included discussion of patient’s health beliefs, providing adherence support, rationalizing therapy for patients, discussing potential or actual adverse drug effects, assessing lifestyle changes, and prompting for medical checks for complications. Visit 1 (recruitment) instructions for blood glucose monitoring, baseline data on diabetes history, quality of life (QoL), well-being, adherence. Visit 2; blood glucose readings, interventions based on identified issues, goals for next visit. Visit 3; blood glucose data, questions regarding lifestyle and self-care, suggestions for change, goal setting. Patients with medication related issues were given a full medication review. Subsequent visits to the pharmacy were tailored to individual needs. | | |
| Frequency of meetings | At least 4 meetings | | |
| Duration of meetings | Not reported | | |
| Intervention team | Pharmacist | | |
| Education intervention team | Yes, education manual and two-day workshop | | |
| Intervention topics | Medication, lifestyle, individual care plan/goal setting, self-management skills, self-monitoring blood glucose, other | | |
| Control group |  | | |
| Description | Usual care. Data was collected at baseline and after 9 months. No blood glucose monitoring as this was considered to be an intervention. | | |
| Outcomes |  | | |
| Clinical outcomes | HbA1c, mean blood glucose | | |
| Patient reported outcomes | QoL (ADDQoL), well-being (WB-Q12), risk of nonadherence (BMQ) | | |
| Results |  | | |
| Clinical results | HbA1c: Statistic significant reduction in intervention group (baseline; 7.9 ± 1.4, 9 months; 7.4 ± 1.3). No change in control group (7.4 ± 1.1). No significant difference between intervention and control group after 9 months.  Blood glucose: Overall significant downward linear trend from visit 1 through 6. | | |
| Patient reported results | QoL: No statistical significant changes.  Well-being: WB-Q12 scores statistical significant increase in intervention group (baseline: 21.9 ± 6.8, 9 months 23.4 ± 6.8) no change in controls (baseline: 21.2 ± 7.3, 9 months 21.2 ± 6.6).  Adherence: Statistical significant reduction of nonadherence in intervention group (baseline: 3.89 ± 1,78, 9 months: 2.74 ± 1.39). Increase in control group (baseline: 2.81 ± 1.15; 9 months: 3.90 ± 1.45). | | |

| Author | Butt (Butt et al., 2015) | | |
| --- | --- | --- | --- |
| Year | 2015 | | |
| Country | Malaysia | | |
| Objective | To evaluate the impact of a pharmacist led diabetes management program on type 2 diabetes patients on HbA1c, medication adherence and quality of life. | | |
| Study setting | Secondary endocrine clinic | | |
| Study design | Parallel randomized controlled trial | | |
| Follow-up period | 6 months | | |
| Sample | N | 73 | |
|  |  | Intervention | Control |
|  | Sex (% male) | 39.4 | 42.4 |
|  | Age (years ± SD) | 57.4 ± 7.2 | 57.1 ± 10.8 |
|  | Baseline HbA1c (% ± SD) | 9.66 ± 1.57 | 9.64 ± 1.41 |
|  | Comorbidities | Not reported | |
| Intervention |  | | |
| Description | Patient Education by Pharmacist Program (PEPP). At enrolment: counselling about diabetes, its complications, medication, lifestyle modifications, and self-monitoring. Second visit; reinforcement of the intervention about the lifestyle modifications, mediation adherence, and self-monitoring. In addition, pharmacist assessed the knowledge of the patients about diabetes and complication components of education and repeated the intervention if the pharmacist felt the need for it after assessment. | | |
| Frequency of meetings | 3 visits | | |
| Duration of meetings | In total 55-75 minutes | | |
| Intervention team | Pharmacist | | |
| Education intervention team | No | | |
| Intervention topics | Diabetes education, medication, lifestyle, self-monitoring blood glucose, other | | |
| Control group |  | | |
| Description | Standard care; patient-physician meeting every 4-9 months. Pharmacy care during prescription refills every 2-3 months. | | |
| Outcomes |  | | |
| Clinical outcomes | HbA1c, fasting blood glucose, lipid profile, BMI | | |
| Patient reported outcomes | Medication adherence (Morisky scale), QoL (EQ5D-3L), diabetes knowledge | | |
| Results |  | | |
| Clinical results | HbA1c: Statistical significant decline in intervention group compared to control group.  BMI: Statistical significant decrease in intervention group, however no significant change between control and intervention group. | | |
| Patient reported results | Medication adherence: Statistical significant improvement in intervention group and compared to control group. Non-significant change within control group.  QoL: Statistical significant change within intervention group for mobility and anxiety as well as for the overall score. Changes in intervention group were statistical significant compared to the changes in the control group. | | |

| Author | Cani (Cani et al., 2015) | | |
| --- | --- | --- | --- |
| Year | 2015 | | |
| Country | Brazil | | |
| Objective | To support informed decision-making, self-care behaviors, problem-solving and active collaboration with the health care team to improve clinical outcomes, health status and quality of life. | | |
| Study setting | Diabetes outpatient clinic | | |
| Study design | Parallel randomized controlled trial | | |
| Follow-up period | 6 months | | |
| Sample | N | 78 | |
|  |  | Intervention | Control |
|  | Sex (% male) | 38.2 | 38.9 |
|  | Age (years ± SD) | 61.9 ± 9.6 | 61.6 ± 8.1 |
|  | Baseline HbA1c (% ± SD) | 9.78 ± 1.55 | 9.61 ± 1.38 |
|  | Comorbidities | Not reported | |
| Intervention |  | | |
| Description | Individualized pharmacotherapeutic care plan (PCP), designed based on necessities identified in the first interview; indication, proper dosage, side effects, storage. Pill organizers were given along with verbal directions on their assembly. Diabetes education; complications, lifestyle changes, regular foot inspections, home blood glucose monitoring. Also written guidance was provided. | | |
| Frequency of meetings | 6 visits | | |
| Duration of meetings | Not reported | | |
| Intervention team | Pharmacist | | |
| Education intervention team | Not reported | | |
| Intervention topics | Diabetes education, medication, lifestyle, self-monitoring blood glucose, other | | |
| Control group |  | | |
| Description | Observed at initial and final assessment. Control patients received standard care. Although they did not receive advice from a clinical pharmacist, they were allowed to request information anytime during the study period. | | |
| Outcomes |  | | |
| Clinical outcomes | HbA1c | | |
| Patient reported outcomes | Self-reported adherence (Morisky-Green questionnaire and Adherence to Medicine Questionnaire (AMQ)), insulin injection, home blood glucose monitoring, QoL (Diabetes Quality of Life Measure), diabetes knowledge. | | |
| Results |  | | |
| Clinical results | HbA1c: Statistical significant decrease within intervention group (baseline; 9.78 ± 1.55, final; 9.21 ± 1.41). No statistical significant difference between control and intervention at final measurement. | | |
| Patient reported results | Statistical significant improvement of diabetes knowledge, medication knowledge, adherence (Morisky-Green), insulin injection technique, home blood glucose monitoring and QoL within the intervention group as well as between control and intervention group at final measurement. | | |

| Author | Choe (Choe et al., 2005) | | |
| --- | --- | --- | --- |
| Year | 2005 | | |
| Country | U.S. | | |
| Objective | To evaluate the effect of case management by a clinical pharmacist on glycemic control and preventive measures in patients with type 2 diabetes. | | |
| Study setting | Ambulatory care clinic | | |
| Study design | Parallel randomized controlled trial | | |
| Follow-up period | 12-24 months | | |
| Sample | N | 80 | |
|  |  | Intervention | Control |
|  | Sex (% male) | 48.8 | 46.1 |
|  | Age (years ± SD) | 52.2 ± 11.2 | 51.0 ± 9.0 |
|  | Baseline HbA1c (% ± SD) | 10.1 ± 1.8 | 10.2 ± 1.7 |
|  | Comorbidities | Not reported | |
| Intervention |  | | |
| Description | Clinical pharmacist as case manager for intervention patients. Therapeutic regiments were evaluated based on efficacy, safety, adverse effects, drug interactions, drug costs and monitoring. Patients had an initial visit with pharmacist (approx. 1 hour), assessment of medication management, basic education regarding diabetes self-management skills (importance of self-care, medication and screening process). Subsequent visits based upon patient’s needs. Monthly telephone calls. Clinical pharmacist periodically reviewed the status of all intervention patients and provided condensed “diabetes status updates” to providers using a standardized form. | | |
| Frequency of meetings | 12 visits/telephone calls | | |
| Duration of meetings | Not reported | | |
| Intervention team | Pharmacist | | |
| Education intervention team | Not reported | | |
| Intervention topics | Medication, self-management skills, other. | | |
| Control group |  | | |
| Description | Controls only received regular care including regular follow-up visits with primary care physicians. No special contact during the intervention and no exit interviews or process measurements at the end of the study. | | |
| Outcomes |  | | |
| Clinical outcomes | HbA1c, LDL, dilated retinal examination, urine micro albumin screening, monofilament testing for diabetic neuropathy. | | |
| Patient reported outcomes | None | | |
| Results |  | | |
| Clinical results | HbA1c: Decrease of HbA1c in both intervention and control group. Statistical significant difference between groups at final measurement.  LDL measurement, retinal examination within 2 years and documented monofilament examination for neuropathy occurred more frequently among those in the intervention group compared with the control group. | | |
| Patient reported results | N/A | | |

| Author | Cohen (Cohen et al., 2011) | | |
| --- | --- | --- | --- |
| Year | 2011 | | |
| Country | U.S. | | |
| Objective | To assess the efficacy of adding a pharmacist-led intensive behavioral and pharmacologic SMA intervention – namely, the Veterans Affairs (VA) Multidisciplinary Education and Diabetes Intervention for Cardiac Risk Reduction-Extended (MEDIC-E) – to standard primary care, as compared to standard primary care alone for the treatment of patients with type 2 diabetes and associated cardiovascular risk factors over a 6 month period. | | |
| Study setting | Veterans affairs medical center | | |
| Study design | Parallel randomized controlled trial | | |
| Follow-up period | 6 months | | |
| Sample | N | 103 | |
|  |  | Intervention | Control |
|  | Sex (% male) | 100 | 96 |
|  | Age (years ± SD) | 69.8 ± 10.7 | 67.2 ± 9.4 |
|  | Baseline HbA1c (% ± SD) | 7.8 ± 1.0 | 8.1 ± 1.4 |
|  | Comorbidities | Heart failure, stroke, coronary heart disease, COPD, mood disorder | |
| Intervention |  | | |
| Description | 4 once-weekly 2-hour sessions followed by 5 monthly booster sessions held in a classroom with approximately 4-6 participants in each session. Sessions consisted of two parts; education in the first half and behavioral and pharmacologic interventions for hypertension, hyperlipidemia, and hyperglycemia and tobacco use in the second half. Topics discussed: diabetes basics, symptomatology, hypertension, dyslipidemia, tobacco use, target goals for each condition, chronic complications, risk factor control, obstacles, solutions, treatment plans for diet, exercise, monitoring and other self-care behaviors. | | |
| Frequency of meetings | First month; 4 weekly meetings. Thereafter 5 monthly meetings. | | |
| Duration of meetings | Weekly visits; 120 minutes  Monthly visits; 90 minutes | | |
| Intervention team | Pharmacist, dietitian, nurse, physical therapist | | |
| Education intervention team | Not reported | | |
| Intervention topics | Diabetes education, medication, lifestyle, individual care plan/goal setting, self-management skills, self-monitoring blood glucose, other. | | |
| Control group |  | | |
| Description | Standard primary care; individual clinic visits with primary care providers once every 4 months. Visits take 20-60 minutes per appointment. | | |
| Outcomes |  | | |
| Clinical outcomes | HbA1c, blood pressure, LDL, HDL, triglycerides, total cholesterol | | |
| Patient reported outcomes | QoL (SF-36 for veterans), 4-question of perceived competence, summary of diabetes self-care activities questionnaire (SDSCA) | | |
| Results |  | | |
| Clinical results | HbA1c: Statistical significant reduction within the intervention group. After 6 months 40.8% of the intervention group achieved target goals for HbA1c compared to 20.4% in the control group.  Blood pressure: Statistical significant reduction of systolic blood pressure (SBP) in the intervention group. After 6 months 58% of the intervention group and 32.7% of the control group achieved goals for SBP. After 6 months there was also a statistical significant difference between intervention and control group.  LDL: At baseline a statistical significant lower level of LDL in the intervention group (96.1 ±25.4 mg/dl) compared to control group (110.7 ± 37.2 mg/dl). After 6 months a statistical significant reduction in the intervention group compared to baseline.  Total cholesterol: At baseline a statistical significant lower level in the intervention group (165.1 ± 34.0 mg/dl) compared to control group (180.7 ± 37.2 mg/dl). | | |
| Patient reported results | QoL: No statistical significant difference from baseline to follow-up in either physical or mental score.  Perceived competence: No statistical significant difference from baseline to follow up.  SDSCA: Statistical significant increase in the number of days per week for following directions for testing blood glucose for both the intervention and control group. Foot care; the number of days of the week that patients followed foot-care recommendations was statistical significant higher in the intervention group but not for the control group. | | |

| Author | Doucette (Doucette et al., 2009) | | |
| --- | --- | --- | --- |
| Year | 2009 | | |
| Country | U.S. | | |
| Objective | To evaluate the effect of community-pharmacist provided extended diabetes care service on primary clinical outcomes; HbA1c, LD-C, BP, and patients’ reported self-care activities. | | |
| Study setting | Community pharmacy | | |
| Study design | Parallel randomized controlled trial | | |
| Follow-up period | 12 months | | |
| Sample | N | 78 | |
|  |  | Intervention | Control |
|  | Sex (% male) | 41.7 | 47.6 |
|  | Age (years ± SD) | 58.7 ± 13.3 | 61.2 ± 10.9 |
|  | Baseline HbA1c (% ± SD) | 7.99 ± 1.45 | 7.91 ± 1.91 |
|  | Comorbidities | Not reported | |
| Intervention |  | | |
| Description | Role pharmacist: gathering information, evaluation the information, formulating a plan, implementing the plan, monitoring the plan, follow-up with patient and physician. During the first visit: pharmacist takes patient’s history, create a medication list, assess clinical markers, review medication and self-care behaviors, and identify drug therapy problems. Subsequent visits were intended to allow pharmacists to follow-up on previous problems, identify new problems, reassess clinical parameters, and discuss self-care activities. | | |
| Frequency of meetings | Up to 4 meetings | | |
| Duration of meetings | Not reported | | |
| Intervention team | Pharmacist | | |
| Education intervention team | Yes, self-study of approximately 15 hours. | | |
| Intervention topics | Medication, individual care plan/ goal setting, self-management skills, other | | |
| Control group |  | | |
| Description | Not reported | | |
| Outcomes |  | | |
| Clinical outcomes | HbA1c, blood pressure, lipid profile | | |
| Patient reported outcomes | Diabetes self-care questionnaire | | |
| Results |  | | |
| Clinical results | HbA1c: Non-significant improvement in intervention group. No significant differences between control and intervention groups.  Blood pressure: Significant increase in intervention group. No significant differences between intervention and control group.  LDL: Statistical significant decrease in both groups. No significant difference between the groups. | | |
| Patient reported results | Diabetes self-care: Statistical significant improvement in intervention group. | | |

| Author | Farsaei (Farsaei et al., 2011) | | |
| --- | --- | --- | --- |
| Year | 2011 | | |
| Country | Iran | | |
| Objective | To evaluate the effect of a clinical pharmacist-led patient education program for type 2 diabetic patients. | | |
| Study setting | Isfahan endocrine and metabolism research center | | |
| Study design | Parallel randomized controlled trial | | |
| Follow-up period | 3 months | | |
| Sample | N | 174 | |
|  |  | Intervention | Control |
|  | Sex (% male) | 36.8 | 31.8 |
|  | Age (years ± SD) | 53.4 ± 9.8 | 52.9 ± 8.5 |
|  | Baseline HbA1c (% ± SD) | 9.3 ± 1.7 | 8.9 ± 1.1 |
|  | Comorbidities | Hypertension, dyslipidemia, heart disease, thyroid disease, renal disease | |
| Intervention |  | | |
| Description | Standard care + pharmacist intervention. Intervention consists of two sessions. First session general extended diabetes education, including difference classifications anti-hyperglycemic agents, dosages, mechanisms of action, indications, efficacy, adverse effects, medication safety issues, contraindications, warnings/precautions, drug interactions, pregnancy risk factors, lactation and storage. Second session: adherence and self-management. After the second session patients received a pill box and diabetes diary log. Individualized patient schedule, medication adherence, dietary adherence, exercise. | | |
| Frequency of meetings | Two education sessions, weekly telephone calls, appointments for glycemic control | | |
| Duration of meetings | Not reported | | |
| Intervention team | Pharmacist | | |
| Education intervention team | Not reported | | |
| Intervention topics | Medication, lifestyle, individual care plan/ goal setting, self-management skills, other | | |
| Control group |  | | |
| Description | Education program offered by nurse; definition of diabetes, diet therapy, controlling measures, symptoms and control of hypo/hyper, and diabetes complications. | | |
| Outcomes |  | | |
| Clinical outcomes | HbA1c, fasting blood glucose | | |
| Patient reported outcomes | None | | |
| Results |  | | |
| Clinical results | HbA1c: Statistical significant reduction in the intervention group.  Fasting blood glucose: Statistical significant reduction in the intervention group. No changes in the control group. | | |
| Patient reported results | N/A | | |

| Author | Jacobs (Jacobs et al., 2012) | | |
| --- | --- | --- | --- |
| Year | 2012 | | |
| Country | U.S. | | |
| Objective | To demonstrate that pharmacists working with physicians in an ambulatory care setting can improve glucose, blood pressure, and lipid control for patients with diabetes type 2. Secondary: whether patients adhered to screening and general preventive measures. | | |
| Study setting | Outpatient clinic | | |
| Study design | Parallel randomized controlled trial | | |
| Follow-up period | 12 months | | |
| Sample | N | 257 | |
|  |  | Intervention | Control |
|  | Sex (% male) | 68 | 55 |
|  | Age (years ± SD) | 62.7 ± 10.8 | 63.0 ± 11.2 |
|  | Baseline HbA1c (% ± SD) | 9.5 ± 1.1 | 9.2 ± 1.0 |
|  | Comorbidities | Retinopathy, nephropathy, neuropathy | |
| Intervention |  | | |
| Description | Patients had to attend a minimum of 3 clinic visits with a clinical pharmacist (baseline; 6 months, 12 months). Intervention content: comprehensive medication review, physical assessment (weight, height, blood pressure, pulse, foot exam), education on diabetes pathophysiology and importance of control, ordering lab tests; reviewing, modifying and monitoring of mediation therapy and providing detailed counselling on all therapies; self-monitoring blood glucose; dietary guidelines and exercise. | | |
| Frequency of meetings | Minimal 3 visits | | |
| Duration of meetings | Not reported | | |
| Intervention team | Pharmacist | | |
| Education intervention team | No | | |
| Intervention topics | Diabetes education, medication, lifestyle, individual care plan/ goal setting, self-management skills, other | | |
| Control group |  | | |
| Description | Usual care directed by physician. Not specified. | | |
| Outcomes |  | | |
| Clinical outcomes | HbA1c (target ≤7%), LDL cholesterol (target ≤100 mg/dl), blood pressure (target ≤ 130/80 mm Hg) | | |
| Patient reported outcomes | None | | |
| Results |  | | |
| Clinical results | HbA1c: Statistical significant decrease in intervention group compared to control (7.7% vs. 8.4%). In intervention group 35% of the participant met the target value (≤ 7%) and 21% in the control group (measured after 12 months). No statistical significant difference between groups.  LDL: Statistical significant decrease in intervention group compared to control group (93.7 vs 105.1 mg/dl). 62% of the intervention group met target values. No statistical significant difference between groups.  Blood pressure: systolic blood pressure (SBP) was significantly higher in intervention group at baseline. SBP decreased in both groups, though no statistical significant difference between groups after 12 months. 51% of intervention an d43% of the control group met target values for SBP. Diastolic blood pressure (DBP) statistical significant decreased more in intervention group than in control group (72.0 vs 77.6 mm Hg). 84% in intervention and 77% in control group met target after 12 months. | | |
| Patient reported results | N/A | | |

| Author | Jahangard-Rafsanjani (Jahangard-Rafsanjani et al., 2015) | | |
| --- | --- | --- | --- |
| Year | 2015 | | |
| Country | Iran | | |
| Objective | To evaluate the effect of a community pharmacist’s diabetes support program on patients with type 2 diabetes receiving specialty care in a middle-income country. | | |
| Study setting | Community pharmacy | | |
| Study design | Parallel randomized controlled trial | | |
| Follow-up period | 5 months | | |
| Sample | N | 101 | |
|  |  | Intervention | Control |
|  | Sex (% male) | 51 | 48 |
|  | Age (years ± SD) | 57.3 ± 8.6 | 55.9 ± 8.7 |
|  | Baseline HbA1c (% ± SD) | 7.6 ± 1.6 | 7.51 ± 1.8 |
|  | Comorbidities | Not reported | |
| Intervention |  | | |
| Description | The program consisted of 5 follow-up visits with the community pharmacist (once a month). Each visit was estimated to be 30 minutes. The community pharmacist made a telephone call between visits to reinforce treatment adherence and resolve any therapy-related problems. Education on diet management, physical activity, diabetes complications. As well as information regarding individual needs. Every follow-up visit medication related problems, self-care issues, and logbook were discussed. Patients were also taught how to self-monitor blood glucose. | | |
| Frequency of meetings | 5 visits | | |
| Duration of meetings | 30 minutes | | |
| Intervention team | Pharmacist | | |
| Education intervention team | Yes | | |
| Intervention topics | Diabetes education, lifestyle, individual care plan/ goal setting, self-management skills, self-monitoring blood glucose, other | | |
| Control group |  | | |
| Description | Usual care from physician. At the end of the study they received brief education from the community pharmacist. | | |
| Outcomes |  | | |
| Clinical outcomes | HbA1c, blood pressure, weight, BMI | | |
| Patient reported outcomes | Adherence (Morisky), self-care activity (SDSCA), physician visits | | |
| Results |  | | |
| Clinical results | HbA1c: Statistical significant reduction in both intervention and control group. No significant difference between groups.  Systolic blood pressure (SBP): No difference within and between groups.  Diastolic blood pressure (DBP): No difference within and between groups.  BMI: No statistical significant difference between groups at baseline, at follow-up BMI statistical significant lower in intervention group compared to control. | | |
| Patient reported results | Adherence: Statistical significant improvement in intervention group (51% to 24%).  Self-care activity: Statistical significant improvement in intervention group for general diet, blood glucose monitoring and foot care.  Physician visits: statistical significant more patients in intervention group visited their physician at least once (71.7% vs. 32.5%). | | |

| Author | Jameson (Jameson and Baty, 2010) | | |
| --- | --- | --- | --- |
| Year | 2010 | | |
| Country | U.S. | | |
| Objective | To investigate the effect of pharmacist management of poor controlled diabetes mellitus in a community base primary care group. | | |
| Study setting | Primary care clinic | | |
| Study design | Parallel randomized controlled trial | | |
| Follow-up period | 12 months | | |
| Sample | N | 104 | |
|  |  | Intervention | Control |
|  | Sex (% male) | 48.9 | 49 |
|  | Age (years ± SD) | 49.3 ± 10.8 | 49.7 ± 10.9 |
|  | Baseline HbA1c (% ± SD) | 10.4 ± 1.2 | 11.1 ± 1.6 |
|  | Comorbidities | Not reported | |
| Intervention |  | | |
| Description | One pharmacist provided all diabetes-relate care for the intervention group. All intervention patients received individualized education regarding diabetes self-management, including diet, exercise, blood glucose level testing, medications, and insulin. The number of subsequent visits was based on the need for further education. Follow-up visits were supplemented with telephone calls as needed for medication management. | | |
| Frequency of meetings | On average 6 visits and 3 telephone calls | | |
| Duration of meetings | Visit: 30-60 minutes. Telephone call: 10-20 minutes. | | |
| Intervention team | Pharmacist | | |
| Education intervention team | No | | |
| Intervention topics | Medication, lifestyle, self-management skills, self-monitoring blood glucose | | |
| Control group |  | | |
| Description | Not reported | | |
| Outcomes |  | | |
| Clinical outcomes | HbA1c (after 1 year and the percentage of patients with a 1.0% decrease) | | |
| Patient reported outcomes | Number of pharmacist visits | | |
| Results |  | | |
| Clinical results | HbA1c: Overall median reduction in the intervention group was 1.1%. Not statistical significant compared to control group. | | |
| Patient reported results | Pharmacist visits: On average 6 pharmacy visits and 3 telephone calls over the course of a year. | | |

| Author | Jarab (Jarab et al., 2012) | | |
| --- | --- | --- | --- |
| Year | 2012 | | |
| Country | Jordan | | |
| Objective | To evaluate the impact of a clinical pharmacist-led pharmaceutical care program on different clinical outcomes and self-management behavior in outpatients with DM2 in Jordan. | | |
| Study setting | Outpatient clinic | | |
| Study design | Parallel randomized controlled trial | | |
| Follow-up period | 6 months | | |
| Sample | N | 171 | |
|  |  | Intervention | Control |
|  | Sex (% male) | 57.6 | 55.8 |
|  | Age (years ± SD) | 63.4 ± 10.1 | 65.3 ± 9.2 |
|  | Baseline HbA1c (% ± SD) | 8.5 | 8.4 |
|  | Comorbidities | Not reported | |
| Intervention |  | | |
| Description | Structured education and discussion considering: diabetes, complications, prescribed drug therapy, dosage, side effects, adherence. Special attentions for lifestyle management (patients were encouraged to change unhealthy diets, perform regularly physical activities, monitoring blood glucose levels). Smoking cessation was discussed. A booklet with all information regarding the education was provided to the patients. During 8 weeks the patients were weekly telephoned to discuss and review the education aspects and answer questions. | | |
| Frequency of meetings | At least 1 visit and 8 telephone calls | | |
| Duration of meetings | Duration of visit unknown, telephone calls approximately 20 minutes. | | |
| Intervention team | Clinical pharmacist | | |
| Education intervention team | Not reported | | |
| Intervention topics | Diabetes education, medication, lifestyle, self-monitoring blood glucose, other | | |
| Control group |  | | |
| Description | Usual care by medical and nursing staff; patient assessment, a 3-6 month review to measure blood glucose, give advice on self-monitoring blood glucose, and nutrition counseling. | | |
| Outcomes |  | | |
| Clinical outcomes | HbA1c, blood pressure, lipid profile, BMI | | |
| Patient reported outcomes | Adherence (Morisky), self-care activity (SDSCA) | | |
| Results |  | | |
| Clinical results | HbA1c: Statistical significant reduction in intervention group and between intervention and control group. In the control group was an increase of HbA1c.  Blood pressure: Statistical significant reduction of blood pressure between the intervention and the control group.  Lipids: No significant improvement in HDL-C levels between the two groups | | |
| Patient reported results | Adherence: No significant difference between groups over the course of the study.  SDSCA: Statistical significant improvement in intervention group for diet, exercise and self-monitoring blood glucose compared to control group. | | |

| Author | Kjeldsen (Kjeldsen et al., 2015) | | |
| --- | --- | --- | --- |
| Year | 2015 | | |
| Country | Denmark | | |
| Objective | To investigate whether a comprehensive and a brief individually targeted intervention for patients with type 2 diabetes could improve implementation of drug therapy in Danish community pharmacies. The intervention intended to give patients more competence and support to improve adherence and self-management in order to reach treatment goals for diabetes and blood pressure as well as goals for patient perceived outcomes. | | |
| Study setting | Community pharmacy | | |
| Study design | Parallel randomized controlled trial | | |
| Follow-up period | 6 months | | |
| Sample | N | 205 | |
|  |  | Intervention | Control |
|  | Sex (% male) | 57.9 (basic intervention), 59.5 (extended intervention) | 62.4 |
|  | Age (years ± SD) | 63 ± 8.8 (basic), 63.4 ± 7.8 (extended) | 62.1 ± 10.2 |
|  | Baseline HbA1c (% ± SD) | Not reported | Not reported |
|  | Comorbidities | Not reported | |
| Intervention |  | | |
| Description | Two interventions were tested; basic intervention (BI) and extended intervention (EI). Key elements for both interventions were; 1. Quick screening for non-adherence and identification of problem types. 2. Patient narratives (story-telling) as the key starting point. 3. Assessment and possibly adjustment of drug therapy. 4. Finding resources in the system around the problem and the patient (the patient’s system). 5. Dialog based on motivational interview or individual coaching, in order to tailor solutions to individual needs and resources. 6. Offering relevant reminder technology and/or patient instruction. 7. Follow-up. 8. Close collaboration with patient’s GP. | | |
| Frequency of meetings | At least 4 visits | | |
| Duration of meetings | In total 65-130 minutes | | |
| Intervention team | Pharmacy assistant and pharmacist | | |
| Education intervention team | Yes | | |
| Intervention topics | Medication, individual care plan/ goal setting, other | | |
| Control group |  | | |
| Description | No information | | |
| Outcomes |  | | |
| Clinical outcomes | Blood glucose, blood pressure, HbA1c, lipid profile | | |
| Patient reported outcomes | Adherence, problems, health related quality of life (HRQoL) (EQ-5D), knowledge about diabetes, perceived competence for diabetes (PCDS), perceived concordance, self-efficacy, hospital admissions, consultations with doctors. | | |
| Results |  | | |
| Clinical results | Blood glucose: Non-significant decrease of blood glucose in both intervention groups.  Blood pressure: Statistical significant decrease of systolic blood pressure within the extended intervention group.  HbA1c: Not enough results for analysis  Lipids: Not enough results for analysis. | | |
| Patient reported results | Concordance: 86% of the participants would like to be actively involved in decision making about their treatment, 85% was satisfied with the decisions made, 55% is sufficiently asked about their treatment.  Knowledge: Statistical significant improvement of knowledge in the EI group, no statistical significant difference between BI and control.  Most frequently delivered technical adherence and self-management improving interventions were self-monitoring of blood glucose, use of diary, use of individual reminder systems, and introduction of dose administration aids. | | |

| Author | Korcegez (Korcegez et al., 2017) | | |
| --- | --- | --- | --- |
| Year | 2017 | | |
| Country | Cyprus | | |
| Objective | To evaluate the effect of a pharmacist-led care program in a public hospital's outpatient diabetes clinic on the clinical outcome of glycemic control, determined primarily by A1c. | | |
| Study setting | Hospital outpatient diabetes clinic | | |
| Study design | Parallel randomized controlled trial | | |
| Follow-up period | 12 months | | |
| Sample | N | 152 | |
|  |  | Intervention | Control |
|  | Sex (% male) | 22.7 | 26.0 |
|  | Age (years ± SD) | 61.80 ±10.38 | 62.22 ±9.54 |
|  | Baseline HbA1c (% ± SD) | 8.29 ±0.89 | 8.31 ±0.84 |
|  | Comorbidities | Hypertension, dyslipidemia, thyroid disease, rheumatoid arthritis, asthma, heart failure, osteoporosis, psychological disorders | |
| Intervention |  | | |
| Description | The pharmacist's face-to-face education and discussion sessions included revision of medication, as necessary, and the sharing of diabetes knowledge, clinical goals and self-care activities. The purpose of the pharmacist-led care program was the educate patients regarding the correct use of medication and reinforce adherence to treatment, along with developing patient knowledge of drug therapy and health conditions. | | |
| Frequency of meetings | 5 meetings in 12 months | | |
| Duration of meetings | NR | | |
| Intervention team | Pharmacist | | |
| Education intervention team | NR | | |
| Intervention topics | Diabetes, medication, lifestyle, medication review, written information, individual care plan/ goal setting, self-management, self-monitoring blood glucose | | |
| Control group |  | | |
| Description | Control group patients met with the research pharmacist at baseline and at the end of 12 months to collect laboratory and questionnaire data. Control group patients received standard care provided by the outpatient diabetes clinic, consisting of appointments with physicians every 4-8 weeks to renew prescriptions for their medicines during the study period. | | |
| Outcomes |  | | |
| Clinical outcomes | Fasting blood glucose, HbA1c, lipid profile, blood pressure, weight | | |
| Patient reported outcomes | Adherence (Morisky Green), self-care (SDSCA) | | |
| Results |  | | |
| Clinical results | Fasting blood glucose: Significant reduction within intervention and control group, no between group differences.  HbA1c: Significant greater reduction in intervention group compared to control (-0.74% vs. -0.04%).  Blood pressure: Systolic- and diastolic blood pressure decreased significantly in intervention group compared to control.  Lipid profile: Significant improvement in total cholesterol in intervention group compared to control group. Significant reduction of HDL-C levels within intervention group. | | |
| Patient reported results | Adherence: Significant improvement within intervention group.  Self-care: Significant improvement in intervention group compared to control for total diet, blood glucose measurement, and foot care. | | |

| Author | Kraemer (Kraemer et al., 2012) | | |
| --- | --- | --- | --- |
| Year | 2012 | | |
| Country | U.S. | | |
| Objective | To determine whether counseling by community pharmacists that provides patient education, goal setting, monitoring, and coaching can better improve patient self-management of diabetes as measured by HbA1c concentrations compared to the distribution of written educational materials without pharmacist counseling. | | |
| Study setting | Outpatient clinic | | |
| Study design | Parallel randomized controlled trial | | |
| Follow-up period | 12 months | | |
| Sample | N | 69 | |
|  |  | Intervention | Control |
|  | Sex (% male) | 61.1 | 38.7 |
|  | Age (years ± SD) | 55.6 ± 6.8 | 52.6 ± 9.2 |
|  | Baseline HbA1c (% ± SD) | 7.28 | 7.38 |
|  | Comorbidities | Not reported | |
| Intervention |  | | |
| Description | Counseling by pharmacist. Pharmacists were trained in gathering patient information, educating and coaching patients and document outcomes. Pharmacists were requested to send a progress note to the GP after each visit. | | |
| Frequency of meetings | On average 5.4 meetings | | |
| Duration of meetings | Not reported | | |
| Intervention team | Pharmacist | | |
| Education intervention team | Yes | | |
| Intervention topics | Individual care plan/goal setting, self-management skills, self-monitoring blood glucose | | |
| Control group |  | | |
| Description | Written education information about managing diabetes and additional information was mailed after 3 months. At the start and end of the study also for the control patients, blood pressure, weight, waist circumference, HbA1c, serum glucose and lipid spectrum were measured. | | |
| Outcomes |  | | |
| Clinical outcomes | HbA1c, lipid profile, fasting blood glucose, blood pressure, weight, waist circumference, BMI | | |
| Patient reported outcomes | The diabetes empowerment scale (DES), The diabetes knowledge test (DKT), Adherence starts with knowledge (ASK-20) | | |
| Results |  | | |
| Clinical results | HbA1c: Statistical significant decrease in intervention group of 0.5% and non-significant decrease of 0.17% in control group. No significant difference between intervention and control group at final follow-up.  HDL-C: Statistical significant decrease in both groups, but no significant decrease between groups.  No statistical significant changes for blood pressure, weight, waist circumference, and BMI. | | |
| Patient reported results | Adherence: No significant changes.  Diabetes empowerment: Statistical significant improvement of scores within the intervention group, no significant differences between intervention and control group. | | |

| Author | Krass (Krass et al., 2007) | | |
| --- | --- | --- | --- |
| Year | 2007 | | |
| Country | Australia | | |
| Objective | To assess the impact of a community pharmacy diabetes service model on patient outcomes in type 2 diabetes. | | |
| Study setting | Community pharmacy | | |
| Study design | Cluster randomized controlled trial | | |
| Follow-up period | 6 months | | |
| Sample | N | 335 | |
|  |  | Intervention | Control |
|  | Sex (% male) | 51 | 51 |
|  | Age (years ± SD) | 62 ± 11 | 62 ± 11 |
|  | Baseline HbA1c (% ± SD) | 8.9 ± 1.4 | 8.3 ± 1.3 |
|  | Comorbidities | Hypertension, high-cholesterol | |
| Intervention |  | | |
| Description | Elements of intervention: Review of self-monitoring of blood glucose; disease, medication and lifestyle education; adherence support and detection of drug-related problems; referrals to GP when appropriate. First visit: blood glucose meter, conversation with patient and additional information supply if needed about adherence, self-management, lifestyle changes regarding weight loss and physical activity. At every meeting individual goals were set and progress discussed during the next meeting. | | |
| Frequency of meetings | 5 visits | | |
| Duration of meetings | Not reported | | |
| Intervention team | Pharmacist | | |
| Education intervention team | Yes | | |
| Intervention topics | Diabetes education, medication, lifestyle, individual care plan/ goal setting, self-management skills, self-monitoring blood glucose, other | | |
| Control group |  | | |
| Description | Usual care, no specialized diabetes service in the pharmacy. | | |
| Outcomes |  | | |
| Clinical outcomes | HbA1c, blood pressure, lipid profile, BMI | | |
| Patient reported outcomes | Quality of life (EQ-5D) | | |
| Results |  | | |
| Clinical results | HbA1c: Decrease in intervention group 0.97% and in control group 0.27%. Statistical significant larger reduction in intervention group compared to control.  Blood glucose: Statistical significant reduction within intervention group from 9.4 mmol/L to 8.5 mmol/L.  Blood pressure: Systolic blood pressure (SBP) in intervention group statistical significant decrease from 143 mm Hg to 137 mm Hg. Diastolic blood pressure (DBP) in intervention group statistical significant decrease from 82 mm Hg to 79 mm Hg.  Changes in SBP and DBP were non-significant compared to control.  Lipid profile: Statistical significant improvement in both groups, but no significant difference between intervention and control group. | | |
| Patient reported results | Quality of life: Statistical significant improvements in quality of life in the intervention group as indicated by increases in ED-5D health-state scale scores. Though changes on EQ-5D showed no significant difference. | | |

| Author | Mehuys (Mehuys et al., 2011) | | |
| --- | --- | --- | --- |
| Year | 2011 | | |
| Country | Belgium | | |
| Objective | To study the effectiveness and sustainability of effects of a community pharmacist intervention in diabetes care. Primary outcome: glycemic control. Secondary outcomes: adherence, knowledge about diabetes and self-management. | | |
| Study setting | Community pharmacy | | |
| Study design | Cluster randomized controlled trial | | |
| Follow-up period | 6 months | | |
| Sample | N | 288 | |
|  |  | Intervention | Control |
|  | Sex (% male) | 51.0 | 53.7 |
|  | Age (years ± SD) | 63 | 62.3 |
|  | Baseline HbA1c (% ± SD) | 7.7 | 7.3 |
|  | Comorbidities | Not reported | |
| Intervention |  | | |
| Description | Protocol defined intervention at the start of the study and each prescription refill visit. Topics discussed; education about type 2 diabetes and complications, education about correct use of oral hypoglycemic agents, medication adherence, healthy lifestyle education (diet, physical exercise, smoking cessation), reminders about annual eye and foot examination. | | |
| Frequency of meetings | Visit at start and at each prescription- refill visit | | |
| Duration of meetings | Not reported | | |
| Intervention team | Pharmacist | | |
| Education intervention team | Yes | | |
| Intervention topics | Diabetes education, medication, lifestyle. | | |
| Control group |  | | |
| Description | Usual care by pharmacist | | |
| Outcomes |  | | |
| Clinical outcomes | Fasting plasma glucose, HbA1c | | |
| Patient reported outcomes | Adherence, knowledge about diabetes (Brief diabetes knowledge test of the Michigan diabetes research and training center), self-management (SDSCA) | | |
| Results |  | | |
| Clinical results | Fasting plasma glucose: Statistical significant reduction in both intervention and control group. No significant difference between groups.  HbA1c: Statistical significant reduction in intervention group and compared to control group. | | |
| Patient reported results | Knowledge: Statistical significant improvement in intervention group and between groups.  Self-management: No improvement in control group. Statistical significant improvement in intervention group for specific diet, physical exercise and foot care. Between group difference for physical exercise and foot care. | | |

| Author | Nascimento (Nascimento et al., 2015) | | |
| --- | --- | --- | --- |
| Year | 2015 | | |
| Country | Portugal | | |
| Objective | To evaluate the improvement on self-care after an intervention based on the management of pharmacotherapy of diabetes associated with therapeutic education in elderly patients following an at home regime. | | |
| Study setting | Diabetes care clinic | | |
| Study design | Parallel randomized controlled trial | | |
| Follow-up period | 6 months | | |
| Sample | N | 90 | |
|  |  | Intervention | Control |
|  | Sex (% male) | 56.8 | 58.1 |
|  | Age (years ± SD) | 74.2 ± 5.4 | 72.3 ± 4.5 |
|  | Baseline HbA1c (% ± SD) | 8.6 ± 1.2 | 8.2 ± 0.7 |
|  | Comorbidities | Hypertension, dyslipidemia, vascular complications | |
| Intervention |  | | |
| Description | Individualized pharmacotherapy management with the analysis of necessity, safety and effectiveness of medications taken by patients. Individualized therapeutic education on diabetes care and especially on the patient’s pharmacotherapy. | | |
| Frequency of meetings | At least 2 visits | | |
| Duration of meetings | Not reported | | |
| Intervention team | Not specified | | |
| Education intervention team | Not reported | | |
| Intervention topics | Medication, self-management skills | | |
| Control group |  | | |
| Description | Standard medical care consultation | | |
| Outcomes |  | | |
| Clinical outcomes | Fasting blood glucose, HbA1c | | |
| Patient reported outcomes | Adherence to drug therapy (self-reported), adherence to self-assessed care (SDSCA) | | |
| Results |  | | |
| Clinical results | Fasting blood glucose: Statistical significant decrease in intervention group compared to control. Intervention 167.4 ± 39.9 mg/dL to 117.3 ± 26.8 mg/dL. Control 162.33 ± 28.0 mg/dL to 142.2 ± 32.9 mg/dL.  HbA1c: Statistical significant decrease in intervention group compared to control. Intervention 8.6 ± 1.2% to 7.7 ± 0.8%. Control 8.2 ± 0.7% to 7.99 ± 0.67%. | | |
| Patient reported results | Adherence to drug therapy: Intervention group 5.6 ± 0.3 to 5.9 ± 0.1. Control group 5.1 ± 0.78 to 5.7 ± 0.3.  Self-assessed care: In the intervention group statistical significant improvements for general diet, specific diet, exercise, blood glucose management. | | |

| Author | Odegard (Odegard et al., 2005) | | |
| --- | --- | --- | --- |
| Year | 2005 | | |
| Country | U.S. | | |
| Objective | To assess the effect of a pharmacist’s intervention on diabetes control as determined by HbA1c. The effects of pharmacist intervention on change in diabetes medication appropriateness and adherence were assessed as secondary outcomes. | | |
| Study setting | University primary care clinic | | |
| Study design | Parallel randomized controlled trial | | |
| Follow-up period | 6-12 months | | |
| Sample | N | 77 | |
|  |  | Intervention | Control |
|  | Sex (% male) | 52 | 64 |
|  | Age (years ± SD) | 51.6 ± 11.6 | 51.9 ± 10.4 |
|  | Baseline HbA1c (% ± SD) | 10.2 ± 0.8 | 10.6 ± 1.4 |
|  | Comorbidities | Not reported | |
| Intervention |  | | |
| Description | Community pharmacist formulated diabetes care plan (DCP) with patient and implements it. Week 1-4: intensive intervention phase, weekly phone call of clinic visit to follow up on DCP, modify as needed, make necessary referrals. Months 1-6: maintenance phase, phone call or clinic visit (weekly or monthly based on needs acuity) to assess DCP progress, reactivate intensive phase (weekly) for new problems or changes in therapy. Months 7-12: resumption of usual care end of clinical pharmacist follow-up. | | |
| Frequency of meetings | On average 2.1 ± 1.0 visits and 4.5 ± 1.9 telephone calls | | |
| Duration of meetings | Visits 30 minutes, telephone calls 10 minutes. | | |
| Intervention team | Pharmacist | | |
| Education intervention team | Not reported | | |
| Intervention topics | Medication, lifestyle, individual care plan/ goal setting, self-management skills, self-monitoring blood glucose, other. | | |
| Control group |  | | |
| Description | Usual care; subjects were instructed to continue normal care with their primary care provider. Diabetes education was not provided during the baseline interview to avoid introducing an intervention for patients in the control group. | | |
| Outcomes |  | | |
| Clinical outcomes | HbA1c | | |
| Patient reported outcomes | Medication appropriateness (MAI), self-reported adherence, contact moments with pharmacist, self-management, diabetes knowledge, quality of life. | | |
| Results |  | | |
| Clinical results | HbA1c: Statistical significant decrease within intervention group; baseline 10.2%, 6-months 8.7% and 12 months 8.2%. No significant difference with control group. | | |
| Patient reported results | Medication appropriateness: No significant change in medication appropriateness after the intervention.  Adherence: Intervention had no effect on adherence. Control patients reported better adherence than intervention patients.  Contact with pharmacist: On average patients had 4.5 ± 1.9 telephone contacts with the pharmacist, taking approximately 10 minutes per call and 2.1 ± 1.0 in-person visits of approximately 30 minutes per visit.  No results reported for self-management, diabetes knowledge and quality of life. | | |

| Author | Samtia (Samtia et al., 2013) | | |
| --- | --- | --- | --- |
| Year | 2013 | | |
| Country | Pakistan | | |
| Objective | To assess the impact of pharmacist-led interventions on glycemic control, medication adherence, disease knowledge, and lifestyle modifications among patients with diabetes in Southern Punjab, Pakistan. | | |
| Study setting | Diabetes clinic | | |
| Study design | Parallel randomized controlled trial | | |
| Follow-up period | 5 months | | |
| Sample | N | 348 | |
|  |  | Intervention | Control |
|  | Sex (% male) | 52.8 | 48.2 |
|  | Age (years ± SD) | 46.1 | 42.3 |
|  | Baseline HbA1c (% ± SD) | 8.51 | 8.54 |
|  | Comorbidities | Not reported | |
| Intervention |  | | |
| Description | Intervention group received predefined specialized care regarding; education of disease including complications, adherence and effect on glycemic control, diet, sensory changes and foot examination, exercise, self-monitoring of blood glucose, control of HbA1c values and fasting blood glucose, smoking cessation. | | |
| Frequency of meetings | At least 2 visits | | |
| Duration of meetings | Not reported | | |
| Intervention team | Pharmacist | | |
| Education intervention team | Not reported | | |
| Intervention topics | Diabetes education, medication, lifestyle, self-management skills, self-monitoring blood glucose, other. | | |
| Control group |  | | |
| Description | Usual medical care | | |
| Outcomes |  | | |
| Clinical outcomes | Fasting blood glucose, HbA1c, BMI waist circumference, blood pressure | | |
| Patient reported outcomes | Adherence, knowledge regarding disease, self-monitoring, life-style modifications. Smoking, education, diabetes type, medication used. | | |
| Results |  | | |
| Clinical results | Fasting blood glucose: Statistical significant improvement in intervention group. No significant difference between intervention and control group.  HbA1c: Statistical significant improvement in intervention group, no significant difference between intervention and control group.  Waist: Statistical significant improvement in the intervention group and between intervention and control group.  BMI: Statistical significant improvement in the intervention group and between intervention and control group. | | |
| Patient reported results | Adherence: No significant differences within intervention and control group, but statistical significant improvement in intervention group compared to control group.  Knowledge and self-care: Statistical significant improvement in the intervention group for “knowledge of sensory changes”, “foot care”, “self-monitoring blood sugar”, “role of exercise” and “dietary restrictions”. Statistical significant improvement between groups in favor of intervention for "knowledge regarding sensory changes", "self-monitoring of blood sugar" and "role of exercise".  Smoking: Statistical significant increase of the percentage of non-smokers in the intervention group. No significant difference between intervention and control group. | | |

| Author | Sarkadi (Sarkadi and Rosenqvist, 2004) | | |
| --- | --- | --- | --- |
| Year | 2004 | | |
| Country | Sweden | | |
| Objective | To investigate the effectiveness of an experience-based group educational program 24 months after baseline and the pinpoint mediators that might play a role in achieving desired metabolic outcomes. | | |
| Study setting | Not reported | | |
| Study design | Parallel randomized controlled trial | | |
| Follow-up period | 12 months + final follow up after 24 months | | |
| Sample | N | 77 | |
|  |  | Intervention | Control |
|  | Sex (% male) | Not reported | Not reported |
|  | Age (years ± SD) | 66.4 | 66.5 |
|  | Baseline HbA1c (% ± SD) | 6.45 | 6.45 |
|  | Comorbidities | Not reported | |
| Intervention |  | | |
| Description | Practical aspects of diabetes management were discussed including; diet, performing self-monitoring tasks, exercise. All participants in the intervention group received a booklet “how to manage your diabetes” with information regarding; logs of imaginary people, information about complications, personal plan for follow-up visits. | | |
| Frequency of meetings | 12 visits | | |
| Duration of meetings | Not reported | | |
| Intervention team | Pharmacist and nurse | | |
| Education intervention team | Yes | | |
| Intervention topics | Diabetes education, medication, lifestyle, self-management skills, self-monitoring blood glucose, other. | | |
| Control group |  | | |
| Description | No intervention for 12 months. Control group received invitation to participate in educational program 24 months after the start of the initial study. | | |
| Outcomes |  | | |
| Clinical outcomes | HbA1c, BMI | | |
| Patient reported outcomes | Personal perception of diabetes | | |
| Results |  | | |
| Clinical results | HbA1c: Statistical significant improvement in intervention group compared to control group after 6 months and 24 months. Control group showed no significant improvement over 24 months. | | |
| Patient reported results | Personal perception: Statistical significant improvement in favour of intervention group compared to control for; “being more satisfied with one’s own knowledge about diabetes”, “exercising more in order to affect blood glucose levels before measurement”, “being able to predict current blood glucose levels before measuring it”. | | |

| Author | Shao (Shao et al., 2017) | | |
| --- | --- | --- | --- |
| Year | 2017 | | |
| Country | China | | |
| Objective | To evaluate the effect of pharmaceutical care on T2DM outpatients | | |
| Study setting | Endocrinology outpatient service | | |
| Study design | Parallel randomized controlled trial | | |
| Follow-up period | 6 months | | |
| Sample | N | 199 | |
|  |  | Intervention | Control |
|  | Sex (% male) | 51.0 | 47.5 |
|  | Age (years ± SD) | 58.86 ±10.59 | 59.20 ±10.34 |
|  | Baseline HbA1c (% ± SD) | 7.38 ±1.71 | 7.37 ±1.44 |
|  | Comorbidities | NR | |
| Intervention |  | | |
| Description | Intervention program included education and interviews. Education consisted of basic knowledge of T2DM, risk of diabetes complications, proper use and precautions of oral antidiabetics and insulin, signs or symptoms of hypoglycemia and self-management, appropriate self-blood glucose monitoring and healthy lifestyle. During the interviews pharmacists discussed with each patient about their medication adherence, self-monitoring of blood glucose, exercise, explained side-effects of drugs and possible drug interactions. | | |
| Frequency of meetings | Two educational sessions (baseline and third month), 3 face-to-face interviews (every other month), 6 telephone interviews (every month) | | |
| Duration of meetings | NR | | |
| Intervention team | Pharmacist | | |
| Education intervention team | NR | | |
| Intervention topics | Diabetes, medication, lifestyle, self-management, self-monitoring blood glucose | | |
| Control group |  | | |
| Description | Control group received usual care from the medical staff and no additional pharmaceutical care from a clinical pharmacist. | | |
| Outcomes |  | | |
| Clinical outcomes | Height, weight, blood pressure, fasting blood glucose, postprandial blood glucose 2h (PBG2h), HbA1c, lipids | | |
| Patient reported outcomes | Adherence (Morisky Green) | | |
| Results |  | | |
| Clinical results | Fasting blood glucose: Significant decrease within intervention group and compared to control group.  HbA1c: Significant decrease within intervention group and compared to control group.  BMI: Significant decrease in both groups, no between group differences.  Blood pressure: Systolic blood pressure decreased significantly within intervention group. Diastolic blood pressure significantly decreased in intervention group and increased in control group.  Lipids: Total cholesterol significantly decreased in intervention group and compared to control group. Triglycerides decreased significantly within intervention group. LDL-C significantly increased in control group. HDL-C significant reduction in intervention group. | | |
| Patient reported results |  | | |

| Author | Taveira (Taveira et al., 2010) | | |
| --- | --- | --- | --- |
| Year | 2010 | | |
| Country | U.S. | | |
| Objective | To assess whether the VA-MEDIC (veterans affairs multi-disciplinary education and diabetes intervention for cardiac risk reduction), a pharmacist-led group medical visit program, could improve achievement of target goals in hypertension hyperglycemia, hyperlipidemia, and tobacco use in patient with type 2 diabetes compared to usual care. | | |
| Study setting | VA (veteran affairs) medical center | | |
| Study design | Parallel randomized controlled trial | | |
| Follow-up period | 4 months | | |
| Sample | N | 118 | |
|  |  | Intervention | Control |
|  | Sex (% male) | 91.4 | 100 |
|  | Age (years ± SD) | 62.2 ± 10.3 | 66.8 ± 10.2 |
|  | Baseline HbA1c (% ± SD) | 8.5 ± 1.5 | 7.9 ± 1.1 |
|  | Comorbidities | Hypertension, hyperlipidemia, coronary heart disease, congestive heart failure | |
| Intervention |  | | |
| Description | 4 group sessions with each 2 education parts; education (40-60 minutes) and behavioral and pharmacologic interventions (60-80 minutes). Topics discussed by pharmacist; diabetes overview, prevention of acute complications, goals setting, use of monitoring equipment, smoking cessation, pharmacological case management, report card and review of this diet and weight loss, physical activity. | | |
| Frequency of meetings | 4 weekly group visits | | |
| Duration of meetings | 120 minutes per visit | | |
| Intervention team | Pharmacist, nutritionist, physical therapist, pharmacist plays a role in 5/8 sessions. | | |
| Education intervention team | Not reported | | |
| Intervention topics | Diabetes education, lifestyle, individual care plan/ goal setting, self-management skills | | |
| Control group |  | | |
| Description | Usual care provided by primary care providers at VA Medical Center | | |
| Outcomes |  | | |
| Clinical outcomes | HbA1c, blood pressure, fasting lipids, BMI | | |
| Patient reported outcomes | Self-care behaviors, tobacco use | | |
| Results |  | | |
| Clinical results | HbA1c: Statistical significant decrease in intervention group (0.9%). Also statistical significant compared to control group.  Blood pressure: systolic blood pressure statistical significant decrease within intervention group. Diastolic blood pressure statistical significant decrease within intervention group and compared to control group.  Lipids: Statistical significant improvement within intervention group.  BMI: No significant changes in intervention and control group. | | |
| Patient reported results | Self-care: Statistical significant improvement in intervention group from 64.7% to 82.6%. Greatest improvement in “blood glucose self-monitoring” and “blood pressure self-monitoring”.  Tobacco use: In the intervention group 3/20 stopped and in control group 0/7 stopped. | | |

| Author | Taveira (Taveira et al., 2011) | | |
| --- | --- | --- | --- |
| Year | 2011 | | |
| Country | U.S. | | |
| Objective | Whether shared medical appointments (SMAs) are feasible for the treatment of diabetes in patients with depression and to evaluate whether these can be efficacious when led by non-physician professionals (e.g. clinical pharmacist with prescribing authority. | | |
| Study setting | Veterans affairs medical hospital | | |
| Study design | Parallel randomized controlled trial | | |
| Follow-up period | 6 months | | |
| Sample | N | 88 | |
|  |  | Intervention | Control |
|  | Sex (% male) | 100 | 95.5 |
|  | Age (years ± SD) | 60.2 ± 9.3 | 61.4 ± 9.9 |
|  | Baseline HbA1c (% ± SD) | 8.3 ± 1.7 | 8.5 ± 1.9 |
|  | Comorbidities | Depression (mandatory for participation), coronary artery disease, anxiety, schizophrenia, bipolar, PTSD | |
| Intervention |  | | |
| Description | VA-MEDIC-D; 4 once weekly and 5 monthly booster sessions. Each session consisted of two parts; education (40-60 minutes) and behavioral and pharmacologic interventions (60-80 minutes). Each session focusses on self-care behavior, nutrition goals, management of daily aspects of diabetes care through discussion, group counseling. Each participant had their own cardiovascular risk report card. Medication changes if needed (expect for depression). Each participant was provided with a individualized homework. | | |
| Frequency of meetings | 4 weekly visits, 4 monthly visits. | | |
| Duration of meetings | Weekly meetings took approximately 120 minutes. No information on the monthly meetings. | | |
| Intervention team | Nurse, nutritionist, clinical pharmacist | | |
| Education intervention team | No | | |
| Intervention topics | Diabetes education, medication, lifestyle, individual care plan/ goal setting, self-monitoring blood glucose and other. | | |
| Control group |  | | |
| Description | Standard diabetes care with primary care provider, approximately 30 minutes. DSME (diabetes self-management education program) consisting of 4 once weekly meetings and monthly follow-up visits. Care provided by pharmacists, nurses and nutritionists. DSME had similar learning objectives as VA-MEDIC-D. | | |
| Outcomes |  | | |
| Clinical outcomes | HbA1c, blood pressure, fasting lipid levels | | |
| Patient reported outcomes | 10 year coronary event risk, depression symptoms, patient health questionnaire (PHQ-9) perceived competence for diabetes (PCDS), diabetes self-care activities (SDSCA), emergency department (ER), smoking, death | | |
| Results |  | | |
| Clinical results | HbA1c: Statistical significant more participants reached guideline HbA1c in intervention group.  Medication: intervention patients were more likely to have medication changes, either in dose increase or initiation of any antihypertensive or antihyperglycemic agent.  No significant differences for blood pressure, lipids and smoking. | | |
| Patient reported results | Coronary risk: Statistical significant decrease of the risk in the intervention group. No significant difference between intervention and control group.  PCDS: No significant difference within and between intervention and control group.  SDSCA: No significant difference within and between intervention and control group.  ER: No significant difference within and between intervention and control group.  No diabetes related admissions or deaths for either group over the course of the study. | | |

| Author | Wishah (Wishah et al., 2015) | | |
| --- | --- | --- | --- |
| Year | 2015 | | |
| Country | Jordan | | |
| Objective | To evaluate the impact of pharmaceutical care interventions on glycemic control and other health-related clinical outcomes in patients with type 2 diabetes. | | |
| Study setting | Outpatient diabetes clinic | | |
| Study design | Parallel randomized controlled trial | | |
| Follow-up period | 6 months | | |
| Sample | N | 106 | |
|  |  | Intervention | Control |
|  | Sex (% male) | 38.5 | 48.1 |
|  | Age (years ± SD) | 52.9 ± 9.6 | 53.2 ± 11.2 |
|  | Baseline HbA1c (% ± SD) | 8.9 ± 1.6 | 8.2 ± 1.3 |
|  | Comorbidities | Not reported | |
| Intervention |  | | |
| Description | Assessment of patient condition. Compose care plan and discuss with physicians. Monitoring of lab results. During meeting with patient; structured patient education and counseling about DM2, medication, side effects, adherence to self-care activities. Also printed information material was provided containing the following information; medication, lifestyle modifications, self-care activities. During every visit to the clinic the patient had a 30 minute session with the pharmacist. Also telephone calls were made to discuss and review the care plan. | | |
| Frequency of meetings | 3 visits | | |
| Duration of meetings | 30 minutes per visit | | |
| Intervention team | Pharmacist | | |
| Education intervention team | Not reported | | |
| Intervention topics | Diabetes education, medication, lifestyle, individual care plan/ goal setting, self-management skills, other | | |
| Control group |  | | |
| Description | Usual care provided by medical and nursing staff | | |
| Outcomes |  | | |
| Clinical outcomes | HbA1c, fasting blood glucose, lipid profile, weight, height, blood pressure | | |
| Patient reported outcomes | Adherence (Morisky scale), self-care (SDSCA), diabetes knowledge (Michigan diabetes knowledge test) | | |
| Results |  | | |
| Clinical results | HbA1c: Statistical significant improvement in both intervention and control groups as well as between groups in favor of the intervention group.  Fasting blood glucose: Improvement in both groups. Statistical significant difference between groups in favor of intervention group.  Lipid profile: Significant improvement in intervention and control groups, but not between groups.  BMI: No significant difference within and between groups. | | |
| Patient reported results | Adherence: Improvement in both intervention and control group. Statistical significant difference between groups in favor of intervention group.  Self-care: Intervention group had statistical significant better scores compared to baseline and compared to control group after 6 months.  Diabetes knowledge: At baseline both control and intervention group had insufficient knowledge. After 6 months statistical significant improvement in intervention group compared to control group. | | |

Supplementary Table 4: Exclusion reasons full text papers

| Author | Reason for exclusion |
| --- | --- |
| Adepu and Ari, 2010 | Not enough information to determine if study fulfilled inclusion criteria |
| Ahmad et al., 2015 | No self-management |
| Bindu Murali et al., 2016 | No ambulatory care setting |
| Borges et al., 2010 | No self-management |
| Buxton et al., 2010 | No peer reviewed research |
| Chan et al., 2012 | No self-management |
| Christie et al., 2014 | No peer reviewed research |
| Cohen et al., 2010 | No RCT |
| Colom, 2011 | Language; Spanish |
| Cranor and Christensen, 2003 | No RCT |
| Elasy et al., 2001 | Gestational diabetes |
| Erka et al., 2017 | No self-management |
| Farsaei et al., 2010 | Duplicate study |
| Fornos Perez et al., 2004 | Language; Spanish |
| Fornos et al., 2006 | No self-management |
| Hassaballa et al., 2015 | Pharmacist plays minor role in intervention |
| Iram et al., 2010 | Not enough information to determine if study fulfilled inclusion criteria |
| Jennings and McAdam Marx, 2012 | No peer reviewed research |
| Krass et al., 2011 | No usual care control group |
| Lyons et al., 2016 | No separate results for diabetes patients |
| MacLean et al., 2012 | Pharmacist plays minor role in intervention |
| Manju et al., 2016 | No self-management |
| Mitchell et al., 2011 | No RCT |
| Moore et al., 2013 | No RCT |
| Ndefo et al., 2017 | No RCT |
| Nielsen et al., 2006 | No RCT |
| Nishita et al., 2013 | Pharmacist plays minor role in intervention |
| Nor Elina et al., 2014 | No peer reviewed research |
| Obarcanin et al., 2015 | No self-management |
| Obreli et al., 2011 | No self-management |
| Obreli –Neto et al., 2011 | No self-management |
| Raji et al., 2002 | No self-management |
| Ramanath and Santhosh, 2011 | Not enough information to determine if study fulfilled inclusion criteria |
| Rothman et al., 2004 | No peer reviewed research |
| Sadur et al., 1999 | Pharmacist plays minor role in intervention |
| Shane-McWhorter et al., 2015 | No RCT |
| Shrader et al., 2013 | No RCT |
| Suppapitiporn et al., 2005 | Not enough information to determine if study fulfilled inclusion criteria |
| Taylor et al., 2003 | No self-management |
| Uehara et al., 2011 | Not enough information to determine if study fulfilled inclusion criteria |


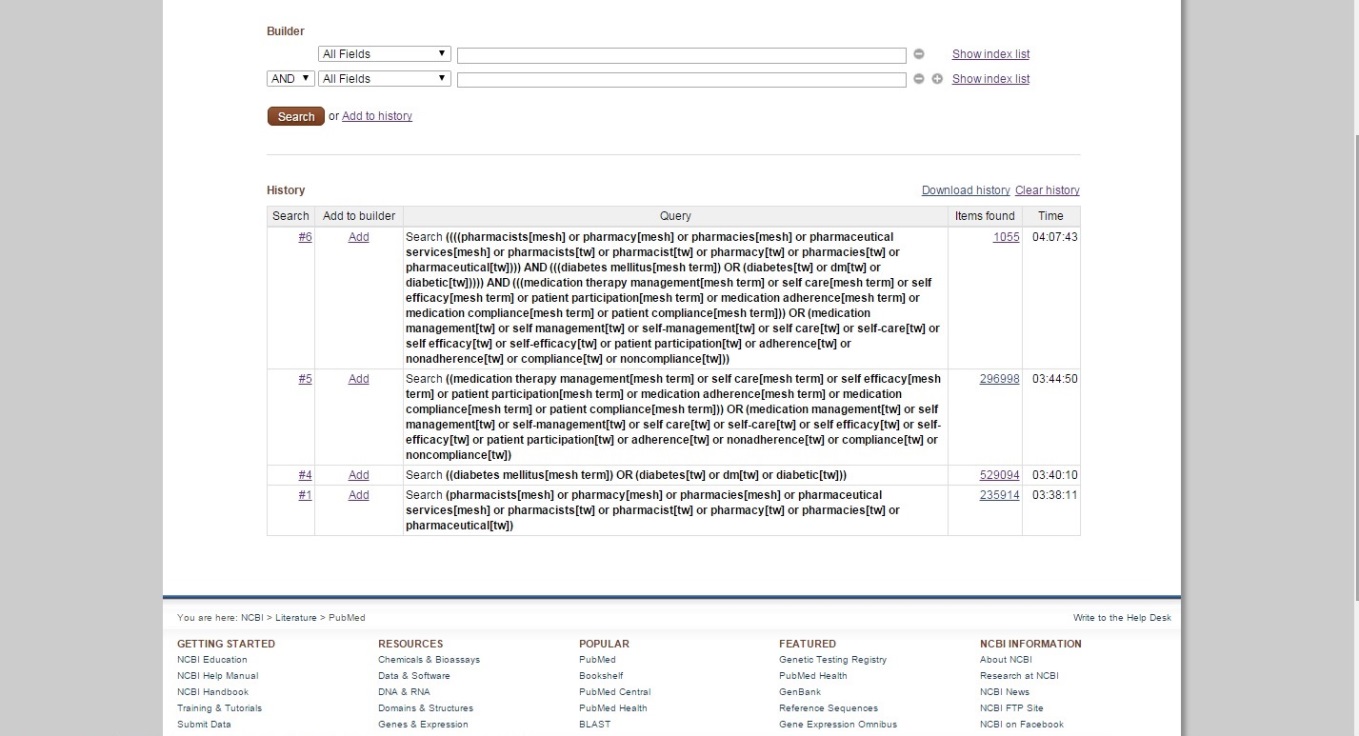


Supplementary Figure 1: Search strategy PubMed


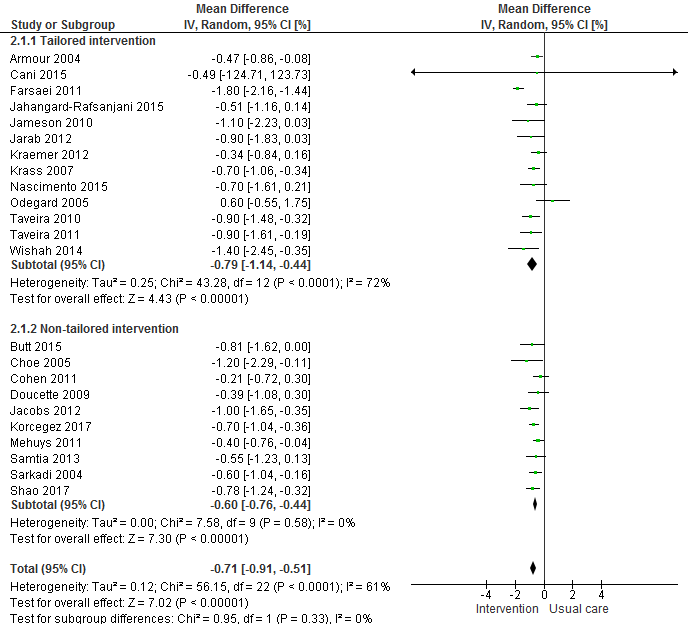


Supplementary Figure 2a: Subgroup analysis tailored intervention


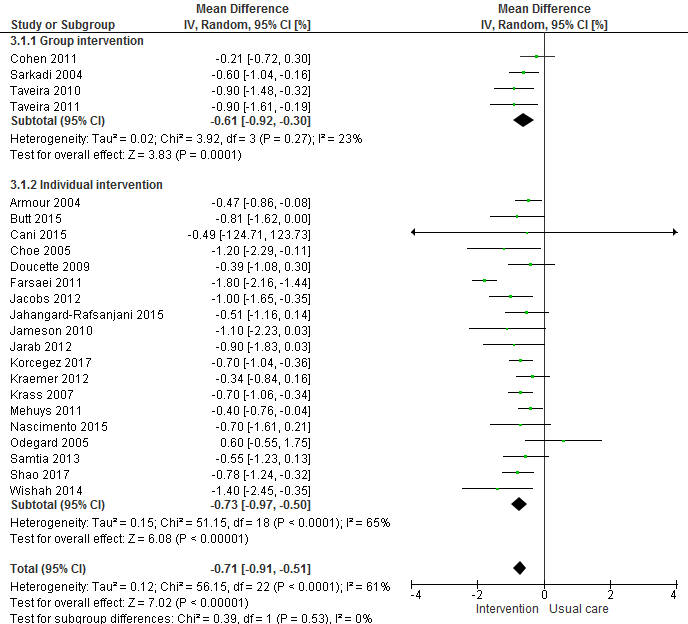


Supplementary Figure 2b: Subgroup analysis Group vs. Individual intervention


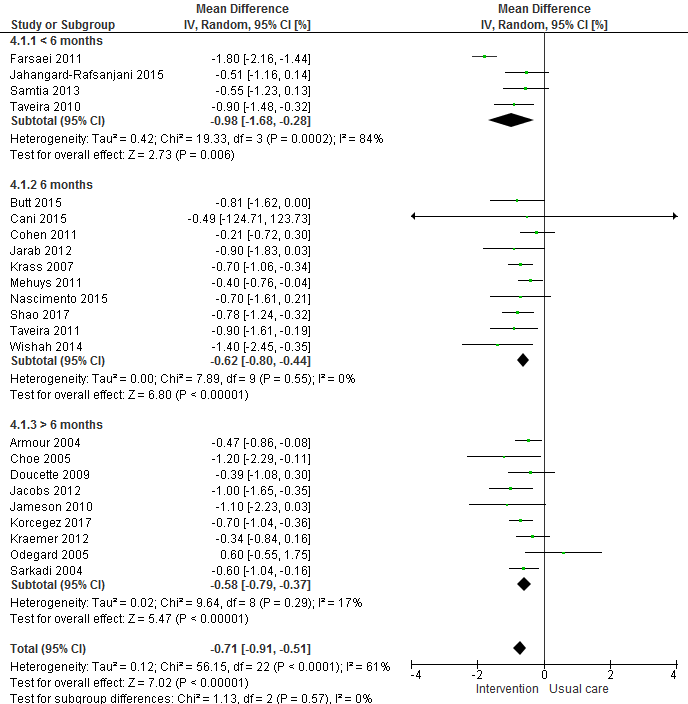


Supplementary Figure 2c: Subgroup analysis <6, 6, >6 months follow-up


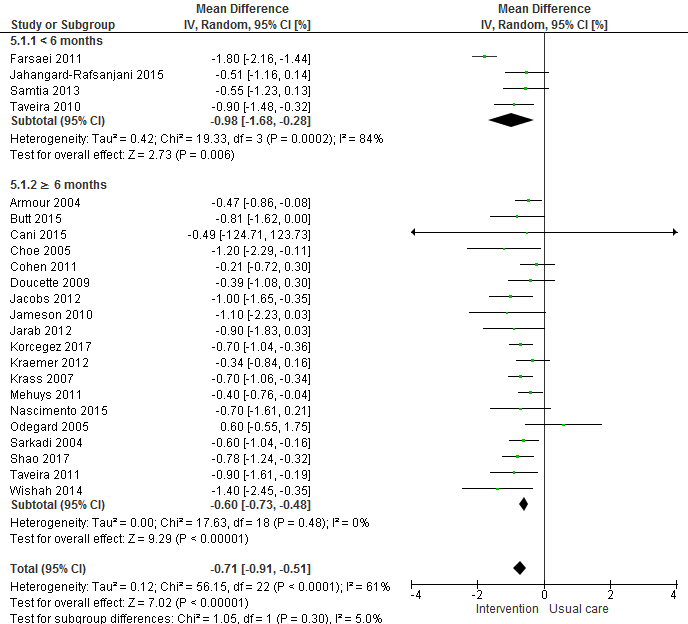


Supplementary Figure 2d: Subgroup analysis <6 months vs. ≥ 6 months follow-up


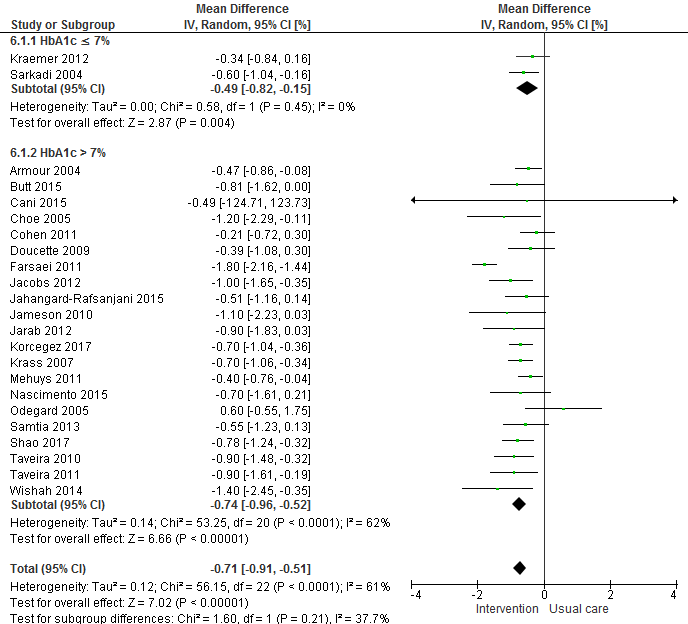


Supplementary Figure 2e: Subgroup analysis baseline HbA1c cut-off 7%


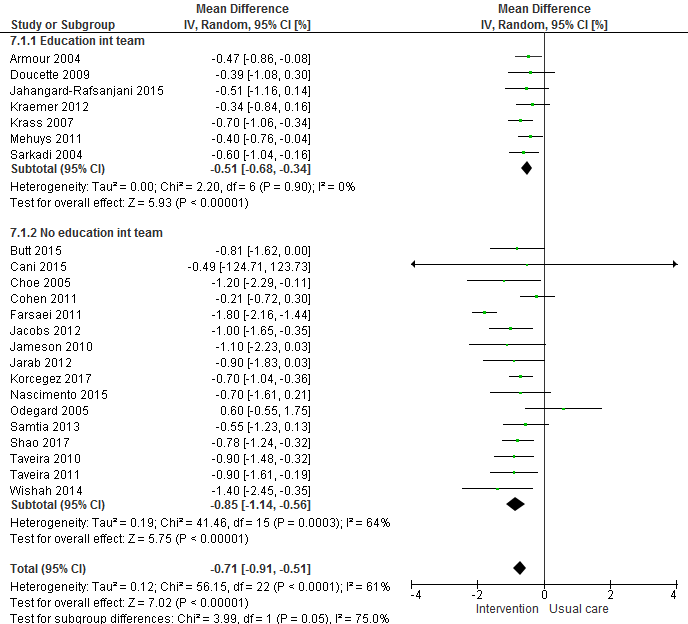


Supplementary Figure 2f: Subgroup analysis education for intervention team


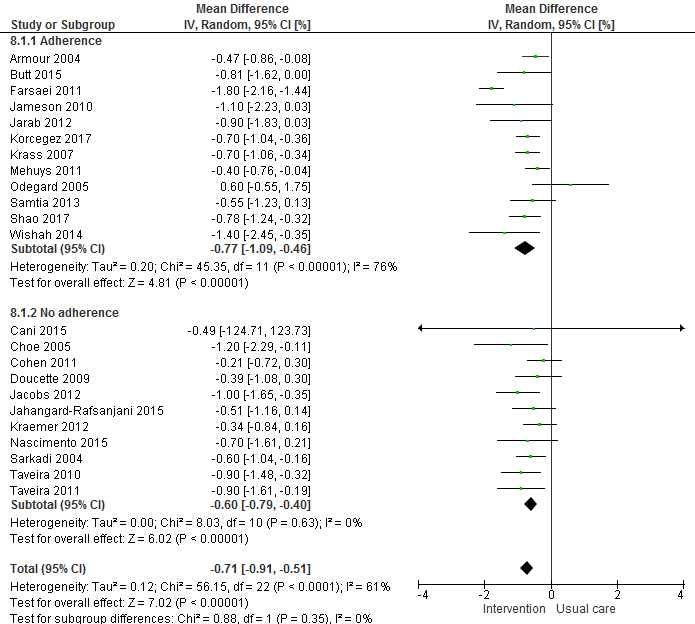


Supplementary Figure 2g: Subgroup analysis Adherence as intervention component


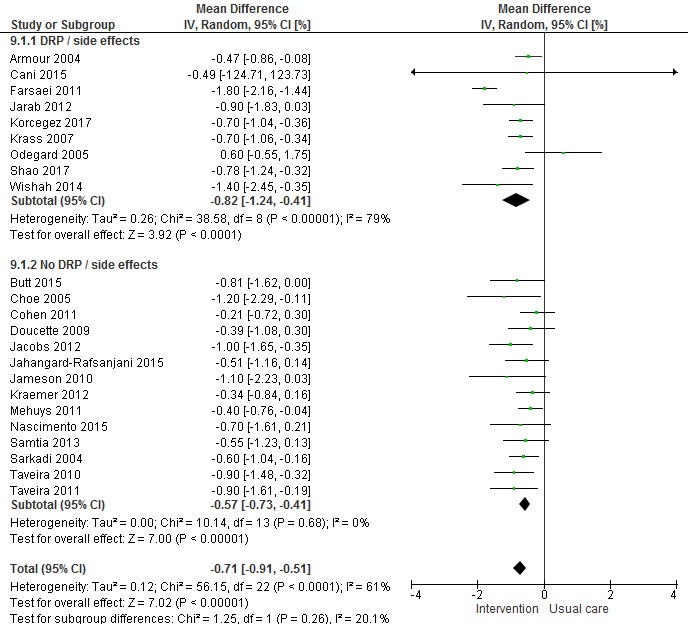


Supplementary Figure 2h: Subgroup analysis DRP/ side effects as intervention component


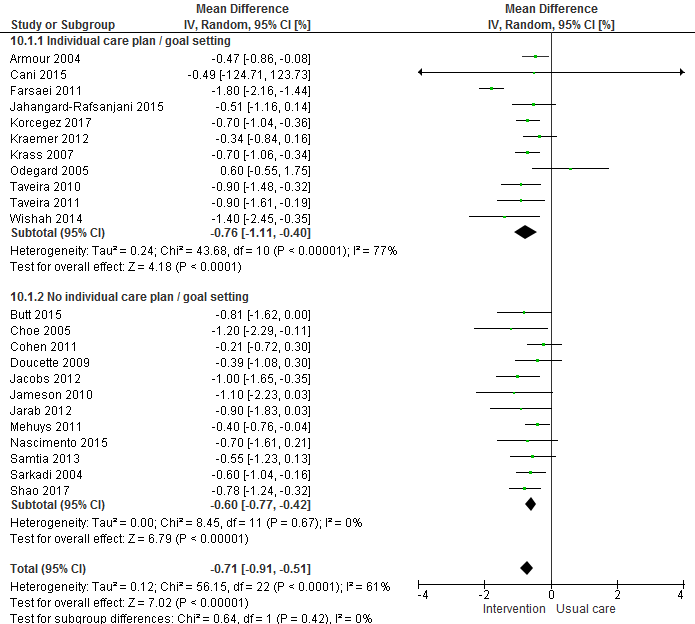


Supplementary Figure 2i: Subgroup analysis individual care plan/ goal setting as intervention component


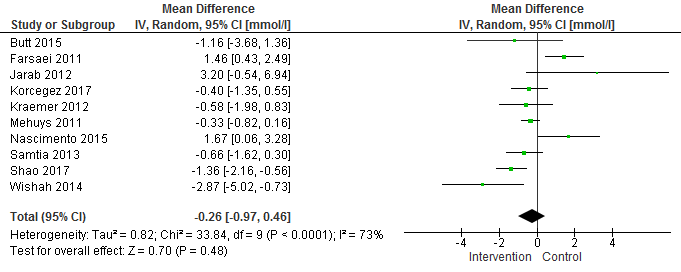


Supplementary Figure 3: Pooled results "Blood Glucose"


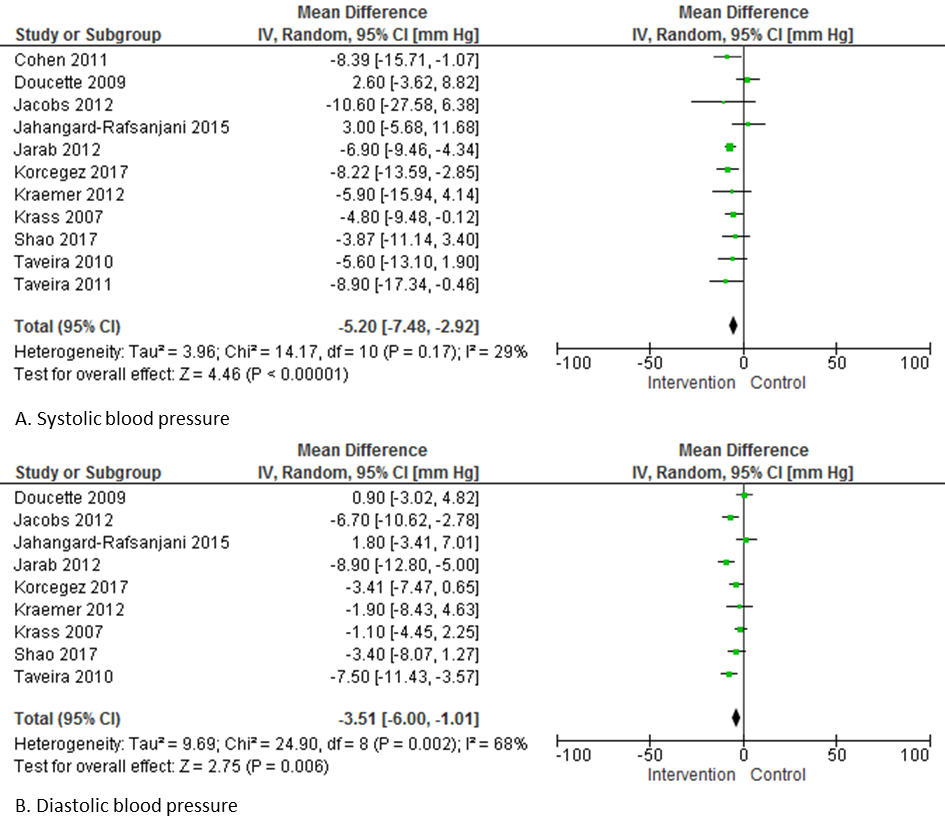


Supplementary Figure 4: Pooled results "Blood Pressure"


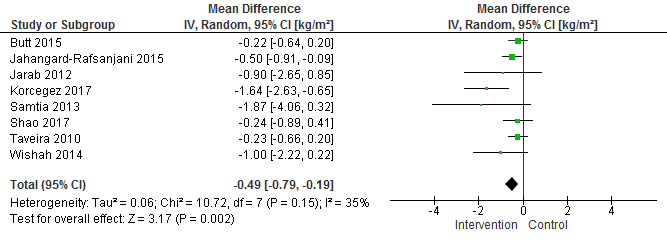


Supplementary Figure 5: Pooled results "BMI"


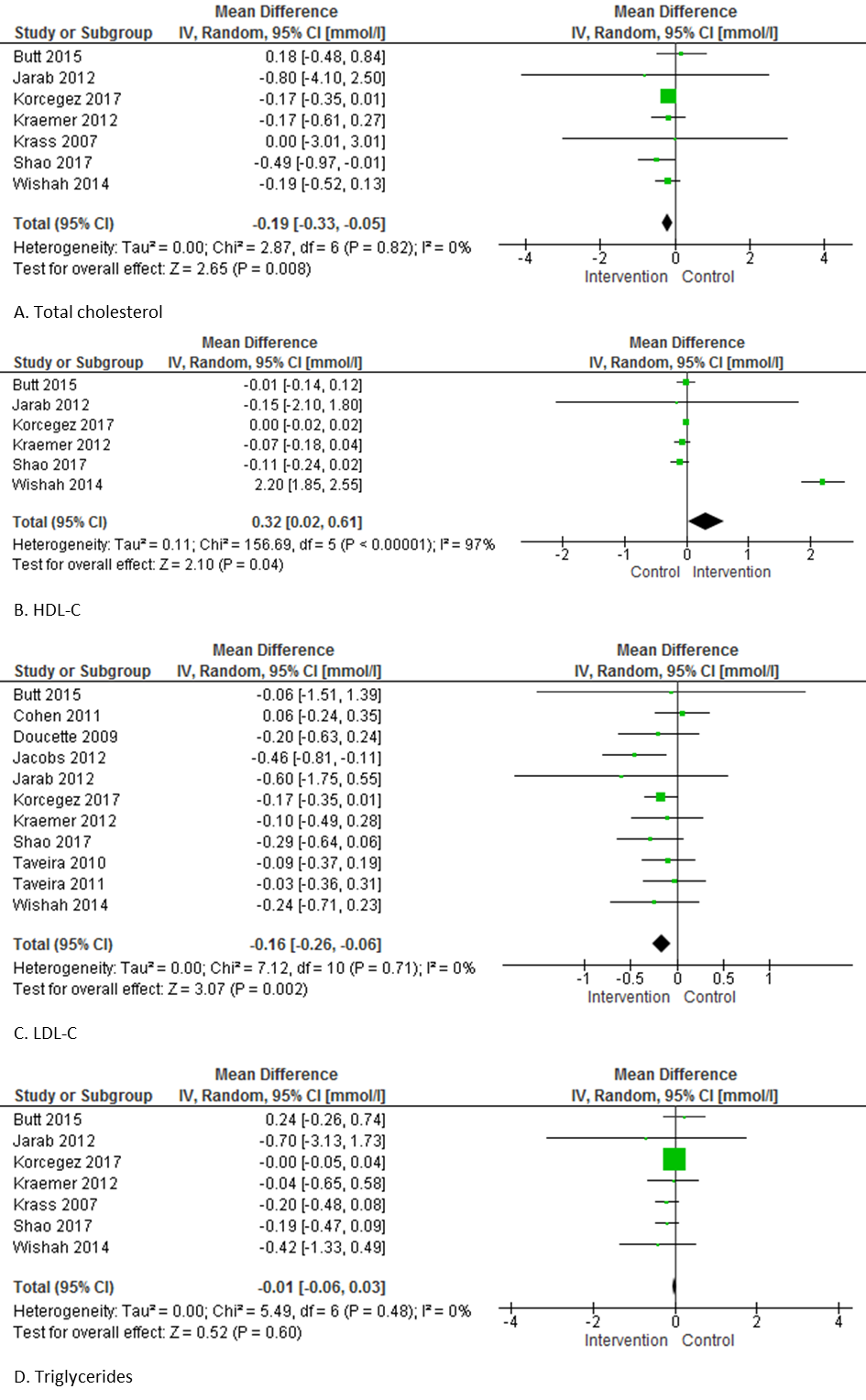


Supplementary Figure 6: Pooled results "Lipids”


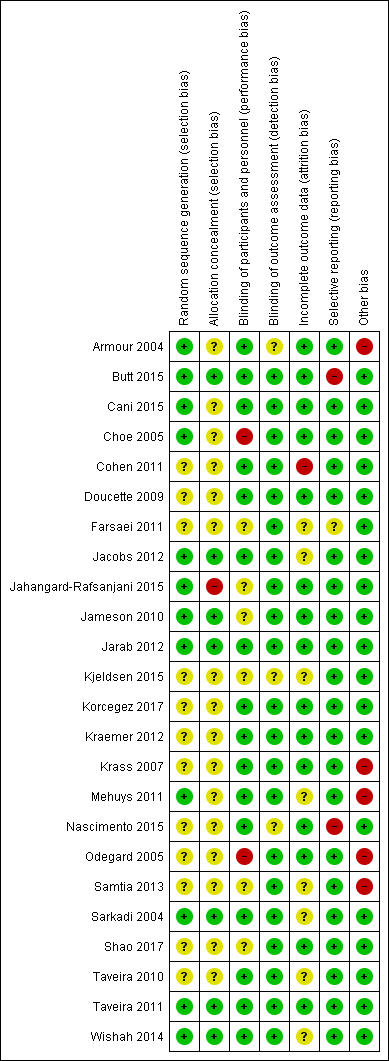


Supplementary Figure 7: Risk of Bias


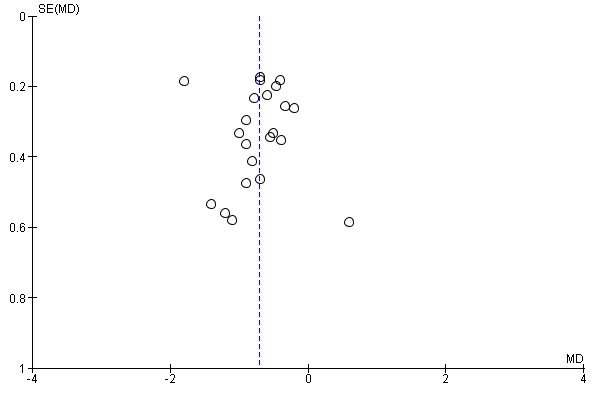


Supplementary Figure 8: Funnel Plot of Publication Bias (Cani et al., 2015 has been left out due to the extremely small SE).

**References**

Adepu, R., and Ari, S.M. (2010). Influence of structured patient education on therapeutic outcomes in diabetes and hypertensive patients. *Asian J Pharm Clin Res.* **3**:3, 174-178.

Ahmad, A.D., Elnour, A.A., Yousif, M.A., Farah, F.H., Akasha, H.A., Abubakar, A. et al. (2015). Pharmacist’s interventions to improve clinical outcomes in patients with type 2 diabetes mellitus: Nyala City, South Darfur State, Sudan. *Int J Diabetes Dev Ctries*. **35**:4, 578-587. doi: 10.1007/S13410-015-0349-5

Armour, C.L., Taylor, S.J., Hourihan, F., Smith, C., and Krass, I. (2004). Implementation and evaluation of Australian pharmacists' diabetes care services. *J Am Pharm Assoc (2003)* **44**:4, 455-466. doi: 10.1331/1544345041475625

Bindu Murali, A., Boban, B., Karoor Shanmughan, A., Marimuthu, K., Remakrishnaneelatha, A., and Xavier, A. (2016). Medication therapy management (MTM): An innovative approach to improve medication adherence in diabetics. *Drug Metab Pers Ther*. **31**:3, 151-155. doi: 10.1515/dmpt-2016-0016

Borges, A.P., Guidoni, C.M., Ferreira, L.D., de Freitas, O., and Pereira, L.R. (2010). The pharmaceutical care of patients with type 2 diabetes mellitus. *Pharm World Sci*. **32:**6, 730-736. doi: 10.1007/s11096-010-9428-3

Butt, M., Mhd Ali, A., Bakry, M.M., and Mustafa, N. (2015). Impact of a pharmacist led diabetes mellitus intervention on HbA1c, medication adherence and quality of life: A randomised controlled study. *Saudi Pharm J*. **24:**1, 40-48. doi: 10.1016/j.jsps.2015.02.023

Buxton, K., Mansell, K., and Jorgenson, D. (2010). Pharmacist interventions: Improving adherence to evidence based therapy. *Can Pharm J*. **143:**2, 74-76.

Cani, C.G., Lopes Lda, S., Queiroz, M., and Nery, M. (2015). Improvement in medication adherence and self-management of diabetes with a clinical pharmacy program: a randomized controlled trial in patients with type 2 diabetes undergoing insulin therapy at a teaching hospital. *Clinics (Sao Paulo)*, **70**:2, 102-106. doi: 10.6061/clinics/2015(02)06

Chan, C.W., Siu, S.C., Wong, C.K., and Lee, V.W. (2012). A pharmacist care program: positive impact on cardiac risk in patients with type 2 diabetes. *J Cardiovasc Pharmacol Ther*. **17:**1, 57-64. doi: 10.1177/1074248410396216

Choe, H.M., Mitrovich, S., Dubay, D., Hayward, R.A., Krein, S.L., and Vijan, S. (2005). Proactive case management of high-risk patients with type 2 diabetes mellitus by a clinical pharmacist: a randomized controlled trial. *Am J Manag Care*. **11:**4, 253-260.

Christie, D., Thompson, R., Sawtell, M., Allen, E., Cairns, J., Smith, F., et al. (2014). Structured, intensive education maximising engagement, motivation and long-term change for children and young people with diabetes: a cluster randomised controlled trial with integral process and economic evaluation – the CASCADE study. *Health Technol Assess*. **18**:20, 1-202. doi: 10.3310/hta18200

Cohen, H.W., Shmukler, C., Ullman, R., Rivera, C.M., and Walker, E.A. (2010). Measurements of medication adherence in diabetic patients with poorly controlled HbA(1c). *Diabet Med*. **27**:2, 210-216. doi: 10.1111/j.1464-5491-2009.02898.x

Cohen, L.B., Taveira, T.H., Khatana, S.A., Dooley, A.G., Pirraglia, P.A., and Wu, W.C. (2011). Pharmacist-led shared medical appointments for multiple cardiovascular risk reduction in patients with type 2 diabetes. *Diabetes Educ*. **37**:6, 801-812. doi: 10.1177/0145721711423980

Colom, F. (2011). Psicoeducación, el litio de las psicoterapias Algunas consideraciones sobre su eficacia y su implementación en la práctica diaria. = Psyco-education, the lithium of psycho-therapies. Some considerations regarding its efficiency and implementation in daily practice. *Revi Colomb Psiquiatr*. **40**, 147S-165S.

Cranor, C.W. and Christensen, D.B. (2003). The Asheville Project: short-term outcomes of a community pharmacy diabetes care program. *J Am Pharm Assoc (Wash)*. **43**:2, 149-159. doi: 10.1331/108658003321480696

Doucette, W.R., Witry, M.J., Farris, K.B., and McDonough, R.P. (2009). Community pharmacist-provided extended diabetes care. *Ann Pharmacother*. **43**:5, 882-889. doi: 10.1345/aph.1L605

Elasy, T., Ellis, S., Brown, A., and Picher,t J. (2001). A taxonomy for diabetes education interventions. *Patient Educ Couns*. **43**:2, 121-127. doi: 10.1016/S0738-3991(00)00150-6

Erku, D.A., Ayele, A.A., Mekuria, A.B., Belachew, S.A., Hailemeskel, B., Tegegn, H.G. (2017). The impacht of pharmacist-led medication therapy management on medication adherence in patients with type 2 diabetes mellitus: a randomized controlled study. *Pharm Pract.* **15**:3, 1026. doi: 10.18549/PharmPract.2017.031026

Farsaei, S., Sabzghabaee, A.M., Zargarzadeh, A.H., and Amini, M. (2010). Effect of pharmacist-led patient education on glycemic control of type 2 diabetics: A randomized controlled trial. *J Res Med Sci*. **15**:6, 317-323.

Farsaei, S., Sabzghabaee, A.M., Zargarzadeh, A.H., and Amini, M. (2011). Effect of pharmacist-led patient education on glycemic control of type 2 diabetics: a randomized controlled trial. *J Res Med Sci* **16**:1, 43-49.

Fornos Perez, J.A., Guerra Garcia, M.M., Andres Rodriguez, N.F., and Egea Ibernon, B. (2004). Evaluation of a programma to monitor drug therapy in type-2 diabetics. *Aten Primaria*. **34**:1, 48-54.

Fornos, J.A., Andres, N.F., Andres, J.C., Guerra, M.M., and Egea, B. (2006). A pharmacotherapy follow-up program in patients with type-2 diabetes in community pharmacies in Spain. *Pharm World Sci*. **28**:2, 65-72. doi: 10.1007/S11096-006-9003-0

Hassaballa, I., Ebekozien, O., Ogungbadero, A., Williams, F., Schultz, J., Hunter-Skidmore, J., Fawcett, S., et al. (2015). Evaluation of a diabetes care coordination program for African-American women living in public housing. *J Clin Outcomes Manag*. **22**:8, 365-372.

Iram, M., Shobha Rani, R.H., and Pais, N. (2010). Impact of patient counselling and education of diabetic patients in improving their quality of life. *Arch Pharm Pract*. **1**:2, 18-22.

Jacobs, M., Sherry, P.S., Taylor, L.M., Amato, M., Tataronis, G.R., and Cushing, G. (2012). Pharmacist Assisted Medication Program Enhancing the Regulation of Diabetes (PAMPERED) study. *J Am Pharm Assoc (2003)*. **52**:5, 613-621. doi: 10.1331/JAPhA.2012.10183

Jahangard-Rafsanjani, Z., Sarayani, A., Nosrati, M., Saadat, N., Rashidian, A., Hadjibabaie, M., et al. (2015). Effect of a community pharmacist-delivered diabetes support program for patients receiving specialty medical care: a randomized controlled trial. *Diabetes Educ*. **41**:1, 127-135. doi: 10.1177/0145721714559132

Jameson, J.P. and Baty, P.J. (2010). Pharmacist collaborative management of poorly controlled diabetes mellitus: a randomized controlled trial. *Am J Manag Care*. **16**:4, 250-255.

Jarab, A.S., Alqudah, S.G., Mukattash, T.L., Shattat, G., and Al-Qirim, T. (2012). Randomized controlled trial of clinical pharmacy management of patients with type 2 diabetes in an outpatient diabetes clinic in Jordan. *J Manag Care Pharm*. **18**:7, 516-526. doi: 10.18553/jmcp.2012.18.7.516

Jennings, B.T. and McAdam Marx, C. (2012). Implementation of a pharmacist-managed diabetes program. *Am J Health Syst Pharm.* **69**:22, 1951-1953. doi: 10,2146/ajhp120252

Kjeldsen, L.J., Bjerrum, L., Dam, P., Larsen, B.O., Rossing, C., Sømdergaard, B., et al. (2015). Safe and effective use of medicines for patients with type 2 diabetes - A randomized controlled trial of two interventions delivered by local pharmacies. *Res Social Adm Pharm*. **11**, 47-62. doi: 10.1016/j.sapharm.2014.03.003

Korcegez, E.I., Sancar, M., Demirkan, K. (2017). Effect of a pharmacist-led program on improving outcomes in patients with type 2 diabetes mellitus from Northern Cyprus: A randomized controlled trial. *J Manag Care Spec Pharm.* **23**:5, 573-582. doi: 10.18553/jmcp.2017.23.5.573

Kraemer, D.F., Kradjan, W.A., Bianco, T.M., and Low, J.A. (2012). A randomized study to assess the impact of pharmacist counseling of employer-based health plan beneficiaries with diabetes: the EMPOWER study. *J Pharm Pract*. **25**:2, 169-179. doi: 10.1177/0897190011418513

Krass, I., Armour, C.L., Mitchell, B., Brillant, M., Dienaar, R., Hughes, J., et al. (2007). The Pharmacy Diabetes Care Program: assessment of a community pharmacy diabetes service model in Australia. *Diabet Med*. **24**:6, 677-683. doi: 10.1111/j.1464-5491.2007.02143.x

Krass, I., Mitchell, B., Song, Y.J.C., Stewart, K., Peterson, G., Hughes, J., et al. (2011). Diabetes Medication Assistance Service Stage 1: impact and sustainability of glycaemic and lipids control in patients with Type 2 diabetes. *Diabetic Med*. **28**:8, 987-993. doi: 10.1111/j.1464-5491.2011.03296.x

Lyons, I., Barber, N., Raynor, D.K., and Wei, L. (2016). The Medicines Advice Service Evaluation (MASE): A randomised controlled trial of a pharmacist-led telephone based intervention designed to improve medication adherence. *BMJ Qual Saf*. **25**:10, 759-769. doi: 10.1136/bmjqs-2015-004670

MacLean, L.G., White Jr,. J.R., Broughton, S., Robinson, J., Schultz, J.A., Weeks, D.L., et al. (2012). Telephone coaching to improve diabetes self-management for rural residents. *Clin Diabetes*. **30**:1, 13-16. doi: 10.2337/diaclin.30.1.13

Manju, C.S., Razak, R., Chandni, R., and Athira, B. (2016). Pharmacist intervention in medication adherence in patients with type II diabetes mellitus. *Int J Pharm Sci Res*. **7**:1, 358-362. doi: 10.13040/ijpsr.0975-8232.7(1).358-62

Mehuys, E., Van Bortel, L., De Bolle, L., Van Tongelen, I., Annemans, L., Remon, J.P., et al. (2011). Effectiveness of a community pharmacist intervention in diabetes care: a randomized controlled trial. *J Clin Pharm Ther*. **36**:5, 602-613. doi: 10.1111/j.1365-2710.2010.01218.x

Mitchell, B., Armour, C., Lee, M., Song, Y.J., Stewart, K., Peterson, G., et al. (2011). Diabetes Medication Assistance Service: the pharmacist’s role in supporting patient self-management of type 2 diabetes (T2DM) in Australia. *Patient Educ Couns*. **83**:3, 288-294. doi: 10.1016/j.pec.2011.04.027

Moore, J.M., Shartle, D., Faudskar, L., Matlin, O.S., and Brennan, T.A. (2013). Impact of patient-centered pharmacy program intervention in a high-risk group. *J Manag Care Pharm*. **19**:3, 228-236. doi: 10.18553/jmcp.2013.19.3.228

Nascimento, T., Braz, N., Gomes, E., Fernandez-Arche, A., and De La Puerta, R. (2015). Self-care improvement after a pharmaceutical intervention in elderly type 2 diabetic patients. *Curr Diabetes Rev*. **12**:2, 120-128.

Ndefo, U.A., Moultry, A.M., Davis, P.N., Askew, R. (2017). Provision of medication therapy management by pharmacists to patients with type-2 diabetes mellitus in a Federally Qualified Health Center. *P T.* **42**:10, 632-637.

Nielsen, A., de Fine Olivarius, N., Gannik, D., Hindsberger, C., and Hollnagel, H. (2006). Structural personal diabetes care in primary health care affects only women’s HbA1c. *Diabetes Care*. **29**:5, 963-969. doi: 10.2337/diacare.295963

Nishita, C., Cardazone, G., Uehare, D.L., and Tom, T. (2013). Empowered diabetes management: life coaching and pharmacist counselling for employed adults with diabetes. *Health Educ Behav*. **40**:5, 581-591. doi: 10.1177/1090198112465088

Nor Elina, A., Che Suraya, M.Z., and Ball, P.A. (2014). The impact of home medication review in patients with type 2 diabetes mellitus living in rural areas of Kuantan, Malaysia. *Value Health*. **17**:3, A127. doi: 10.1016/j.jval.2014.03.736

Obarcanin, E., Krüger, M., Müller, P., Nemitz, V., Schwender, H., Hasanbegovic, S., et al. (2015). Pharmaceutical care of adolescents with diabetes mellitus type 1: the DIADEMA study, a randomized controlled trial. *Int J Clin Pharm*. **37**:5, 790-798. doi: 10.1007/s11096-015-0122-3

Obreli-Neto, P.R., Marusic, S., de Lyra, D.P., Pilger, D., Cruciol-Souza Jr, J.M., Gaeti, W.P., et al. (2011). Effect of a 36-month pharmaceutical care program on coronary heart disease risk in elderly diabetic and hypertensive patients. *J Pharm Pharm Sci*. **14**:2, 249-263.

Obreli-Neto, P.R., Guidoni, C.M., de Oliveira Baldoni, A., Pilger, D., Cruciol-Souza, J.M., Gaeti-Franco, W.P., et al. (2011). Effect of a 36-month pharmaceutical care program on pharmacotherapy adherence in elderly diabetic and hypertensive patients. *Int J Clin Pharm*. **33**:4, 642-649. doi: 10.1007/s11096-011-9518.x

Odegard, P.S., Goo, A., Hummel, J., Williams, K.L., and Gray, S.L. (2005). Caring for poorly controlled diabetes mellitus: a randomized pharmacist intervention. *Ann Pharmacother*. **39**:3, 433-440. doi: 10.1345/aph1E438

Raji, A., Gomes, H., Beard, J., MacDonald, P., and Conlin, P. (2002). A randomized trial comparing intensive and passive education in patients with diabetes mellitus. *Arch Intern Med*. **162**:11, 1301-1304. doi: 10.1001/archinte.162.11.1301

Ramanath, K.V. and Santhosh, Y.L. (2011). Impact of clinical pharmacist provided patient education on QOL outcome in type II diabetes mellitus in rural population. *Asian J Pharm Clin Res*. **4**:4, 15-20.

Rothman, R.L., DeWalt, D.A., Malone, R., and Persell, S.D. (2004). Diabetes disease management program is more effective for patients with low literacy. *J Clin Outcomes Manag.* **11**:12, 752-753.

Sadur, C.N., Moline, N., Costa, M., Michalik, D., Mendlowitz, D., Roller, S., et al. (1999). Diabetes management in a health maintenance organization. Efficacy of care management using cluster visits. *Diabetes Care*. **22**:12, 2011-2017. doi: 10.2337/diacare.22.12.2011

Samtia, A.M., Rasool, M.F., Ranjha, N.M., Usman, F., and Javed, I. (2013). A multifactorial intervention to enhance adherence to medications and disease-related knowledge in type 2 diabetic patients in Southern Punjab, Pakistan. *Trop J Pharm Res*. **12**:5, 851-856. doi: 10.4314/tjpr.v12i5.28

Sarkadi, A. and Rosenqvist, U. (2004). Experience-based group education in Type 2 diabetes: a randomised controlled trial. *Patient Educ Couns*. **53**:3, 291-298. doi: 10.1016/j.pec.2003.10.009

Shane-MacWhorter, L., McAdam-Marx, C., Lenert, L., Peterson, M., Woolsey, S., Coursey, J.M., et al. (2015). Pharmacist-provided diabetes management and education via a telemonitoring program. *J Am Pharm Assoc (2003)*. **55**:5, 516-526. doi: 10.1331/JAPhA.2015.14285

Shao, H., Chen, G., Zhu, C., Chen, Y., Liu, Y., He, Y., et a. (2017). Effect of pharmaceutical care on clinical outcomes of outpatients with type 2 diabetes mellitus. *Patient Prefer Adherence.* **11**, 897-903. Doi: 10.2147/PPA.S92533.

Shrader, S.P., Martin, A., and Cogdill, B. (2013). Effect of group diabetes self-management education classes on clinical outcomes and patient satisfaction in a family medicine clinic. *J Pharm Technol*. **29**:1, 35-39.

Suppapitiporn, S., Chindavijak, B., and Onsanit, S. (2005). Effect of diabetes drug counseling by pharmacist, diabetic disease booklet and special medication containers on glycemic control of type 2 diabetes mellitus: a randomized controlled trial. *J Med Assoc Thai*. **88**: Suppl4, S134-S141.

Taveira, T.H., Friedmann, P.D., Cohen, L.B., Dooley, A.G., Khatana, S.A.M., Pirraglia, P.A., et al. (2010). Pharmacist-led group medical appointment model in type 2 diabetes. *Diabetes Educ*. **36**:1, 109-117. doi: 10.1177/014521709352383

Taveira, T.H., Dooley, A.G., Cohen, L.B., Khatana, S.A.M., and Wu, W. (2011). Pharmacist-Led Group Medical Appointments for the Management of Type 2 Diabetes with Comorbid Depression in Older Adults. *Ann Pharmacother*. **45**:11, 1346-1355. doi: 10.1345/aph.1Q212

Taylor, C.T., Byrd, D.C., and Krueger, K. (2003). Improving primary care in rural Alabama with a pharmacy initiative. *Am J Health Syst Pharm*. **60**:11, 1123-1129.

Uehara, D.L., Nishita, D.M., Tom, T., and Fukunaga, L. (2011). The influence of individualised supports on the self-efficacy of employed diabetics in Hawai’i: Findings from a mixed method study. *J Pac Rim Psychol*. **5**:2, 65-74. doi: 10.1017/s1834490900000593

Wishah, R.A., Al-Khawaldeh, O.A., and Albsoul, A.M. (2015). Impact of pharmaceutical care interventions on glycemic control and other health-related clinical outcomes in patients with type 2 diabetes: Randomized controlled trial. *Diabetes Metab Syndr*. **9**:4, 271-276. doi: 10.1016/j.dsx.2014.09.001
